# Architecture of eukaryotic mRNA 3′-end processing machinery

**DOI:** 10.1126/science.aao6535

**Published:** 2017-10-26

**Authors:** Ana Casañal, Ananthanarayanan Kumar, Chris H. Hill, Ashley D. Easter, Paul Emsley, Gianluca Degliesposti, Yuliya Gordiyenko, Balaji Santhanam, Jana Wolf, Katrin Wiederhold, Gillian L. Dornan, Mark Skehel, Carol V. Robinson, Lori A. Passmore

**Affiliations:** 1 MRC Laboratory of Molecular Biology, Cambridge, UK; 2 Chemistry Research Laboratory, University of Oxford, Oxford, UK; * These authors contributed equally to this work

## Abstract

Newly transcribed eukaryotic precursor messenger RNAs (pre-mRNAs) are processed at their 3’ ends by the ~1-megadalton multiprotein cleavage and polyadenylation factor (CPF). CPF cleaves pre-mRNAs, adds a polyadenylate tail, and triggers transcription termination, but it is unclear how its various enzymes are coordinated and assembled. Here, we show that the nuclease, polymerase, and phosphatase activities of yeast CPFare organized into threemodules. Using electron cryomicroscopy, we determined a 3.5-angstrom-resolution structure of the ~200-kilodalton polymerase module. This revealed four β propellers, in an assembly markedly similar to those of other protein complexes that bind nucleic acid. Combined with in vitro reconstitution experiments, our data show that the polymerase module brings together factors required for specific and efficient polyadenylation, to help coordinate mRNA 3′-end processing.

Protein-coding genes in eukaryotes are transcribed by RNA polymerase II (Pol II) as precursor messenger RNAs (mRNAs), which undergo 5’ capping, splicing, and 3′-end processing. The 3′-end processing machinery includes the highly conserved cleavage and polyadenylation factor (CPF in yeast, CPSF in metazoans) (table S1). CPF interacts with Pol II during transcription, monitoring the nascent pre-mRNA until it recognizes specific RNA elements. It is reported to contain 15 different subunits, including the Ysh1/CPSF73 endonuclease that cleaves the pre-mRNA, the Pap1/PAP polymerase that adds the polyadenylate [poly(A)] tail, and two protein phosphatases (Ssu72/SSU72 and Glc7/PP1) that regulate transcription and 3′-end processing (*1**–**7*). The poly(A) tail is required for nuclear export, confers stability to the mRNA, and regulates translation. Defects in 3′-end processing occur in human diseases, including b-thalassemia, thrombophilia, and cancer, as well as viral infections (*8**–**10*).

Using a tandem affinity purification tag, we purified native CPF from yeast (Fig. 1A) that was active and specific in cleavage and polyadenylation assays: CPF cleaves a model *CYC1* pre-mRNA in vitro and adds a poly(A) tail onto the 5’ (but not 3′) cleavage product (Fig. 1B and fig. S1).

**Fig. 1:**
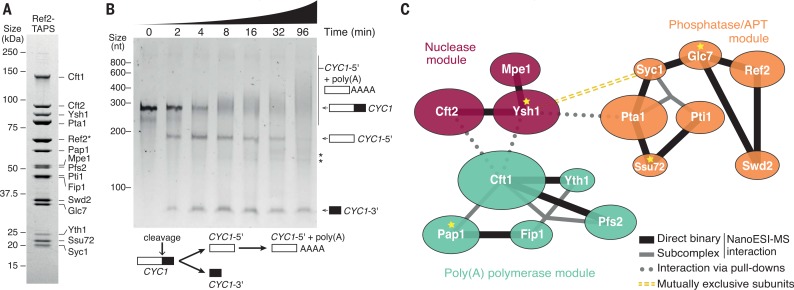
**Architecture of native yeast CPF. (A)** Coomassie-stained SDS-polyacrylamide gel electrophoresis (PAGE) showing CPF/APTpurified from a yeast strain containing TAPS-tagged Ref2 (marked with an asterisk). **(B)** Coupled cleavage and polyadenylation assay of purified CPF analyzed by denaturing urea-PAGE. *CYC1* is the substrate RNA. Cleavage products are *CYC1*-5’ and *CYC1*-3′. Aberrant cleavage products are marked with asterisks. **(C)** Interaction network between subunits of CPF and APT complexes from computational analysis of nanoESI-MS data (solid lines) and from pull-down assays (dotted lines). Black lines indicate confirmed binary interactions; gray lines designate interacting proteins where the direct interaction partner could not be confirmed (see materials and methods). The yellow dashed line denotes that Syc1 and Ysh1 likely bind Pta1 in a mutually exclusive manner. Protein symbols are scaled to have an area proportionate to their molecular weights. Yellow stars denote enzymes.

To understand the architecture of CPF, we analyzed the overall stoichiometry, protein–protein interactions, and composition of the purified complex using noncovalent nanoelectrospray ionization mass spectrometry (nanoESI-MS) (*11*). Ionization causes fragmentation of CPF into 38 different subcomplexes composed of combinations of the 15 previously identified subunits of CPF (table S2). Computational analysis revealed a protein–protein interaction network in which subunits are organized into three prominent modules centered around the CPF enzymatic activities: nuclease ( Ysh1), phosphatase (Ssu72, Glc7), and polymerase (Pap1) (Fig. 1C).

The nuclease module is composed of three subunits (Ysh1, Cft2, and Mpe1), whereas the phosphatase module contains seven subunits (Pta1, Ref2, Pti1, Swd2, Glc7, Ssu72, and Syc1). Syc1 was only observed when CPF was purified from a yeast strain with a tagged phosphatase module subunit (table S2). Syc1 bears homology to the C terminus of Ysh1, and they may occupy the same, mutually exclusive binding site on Pta1 (*12*). Thus, CPFmight contain 14, not 15, subunits, and APT [associated with Pta1 (*5*)] may be a separate complex with six overlapping subunits.

The poly(A) polymerase module contains five subunits: Cft1, Pfs2, Pap1, Fip1, and Yth1 (Fig. 1C). Cft1 appears to play a central role as it is present in 14 of the 15 polymerase module subcomplexes (table S2). Pap1 and Fip1 can be present in up to two copies within the complex (fig. S2A). Fip1 is thought to tether Pap1 to CPF (*13**–**16*). We also observed subcomplexes that contain Pap1, but not Fip1 (table S2), suggesting that Pap1 may contact other CPF subunits (*16**,* *17*). The poly(A) polymerase module is analogous to a four-subunit mammalian complex, which is necessary and sufficient for specific in vitro polyadenylation (*18*). This suggests that the architecture of yeast and mammalian complexes is highly similar.

NanoESI-MS did not reveal interactions between the three enzymatic modules of CPF. The modules may be held together by hydrophobic interactions that are stable in solution but weakened in the gas phase (*11*). Pull-down assays with subunits from each module revealed potential connections between them (fig. S2B).

To understand the molecular basis of subunit association, we used electron cryomicroscopy (cryo-EM) to study CPF isolated from yeast. This resulted in a three-dimensional (3D) reconstruction at ~12 Å resolution (fig. S3). At this resolution, it is not possible to assign densities to subunits. Moreover, this structure is too small to represent the entire CPF complex.

Next, because of the central role of polyadenylation in 3′-end processing, we developed a strategy to overexpress the polymerase module in insect cells for structural and biochemical characterization. This could be purified with or without the Pap1 subunit, consistent with nanoESI-MS (fig. S4A). We imaged the ~200-kDa recombinant four-subunit complex (Cft1, Pfs2, Yth1, Fip1) using cryo-EM (fig. S4, B and C). We determined a 3D reconstruction of the complex, at an overall resolution of 3.5 Å, allowing us to build atomic models into the density for 1717 amino acids (table S3 and figs. S4 and S5). Prior to this, the only highresolution structure available for this complex was a crystal structure of 72 amino acids of CPSF30/ Yth1 (19). The polymerase module is markedly similar to the structure we obtained with the native CPF preparation (fig. S3).

In our cryo-EM map (Fig. 2 and movie S1), three of the four subunits are well ordered: Cft1 (residues 1 to 1357), Pfs2 (residues 27 to 411), and Yth1 (residues 1 to 94). The C-terminal half of Yth1 (zinc fingers 3 to 5, residues 95 to 208), all of Fip1, and several loops in Cft1 are not visible and are presumably disordered, consistent with predictions (fig. S6A).

**Fig. 2:**
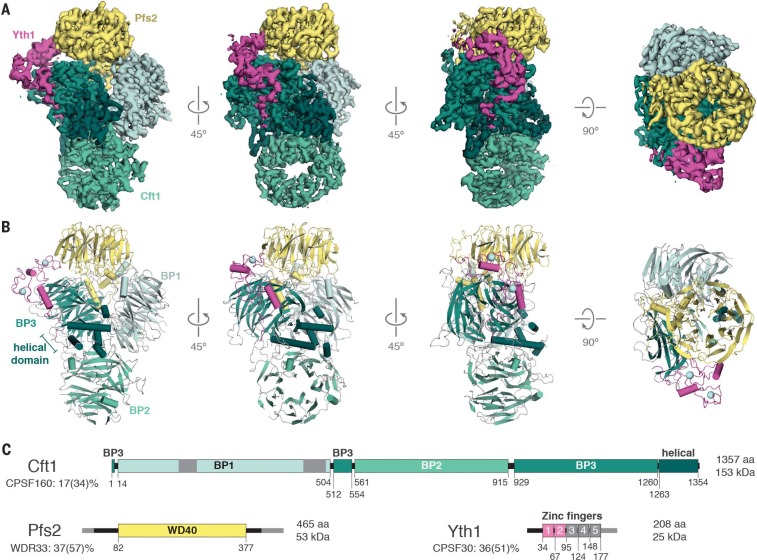
**Cryo-EM of the polymerase module of CPF. (A)** Cryo-EM map and **(B)** cartoon representation of the atomic model of the Cft1–Pfs2–Yth1 complex of the polymerase module. Yth1 (magenta), Pfs2 (yellow), Cft1 (green), and zinc ions (pale cyan) are depicted.The three b-propeller domains of Cft1 (BP1, BP2, and BP3) are colored in different shades. **(C)** Schematic representation of polymerase module subunits present in the cryo-EMstructure, with domain boundaries. Gray regions are not ordered in the cryo-EM map. Human orthologs are given with the percent sequence identity (similarity).

Cft1 forms the core of the complex and is composed of three seven-bladed β propellers followed by a C-terminal helical domain. β propeller 1 (BP1) and BP2 are each formed of contiguous sequences. By contrast, BP3 is predominantly C-terminal, but it also contains one β strand from the N terminus and three β strands from the middle of the protein, creating an intertwined and rigid structural core (fig. S6B). The C-terminal helical domain of Cft1 is located at the nexus of the three β propellers, further stabilizing the fold (Fig. 2B).

Our structure reveals an extensive interface between Cft1 and Pfs2, burying >4200 Å^2^ surface area (Fig. 3A). Almost 50 amino acids in the N-terminal region of Pfs2 are inserted into the cavity between Cft1-BP1 and -BP3, forming contacts with the tops of both β propellers (Fig. 3B). A Pfs2 β propeller (fig. S6C) is then positioned on the top of Cft1 stabilized by loops extending from BP1 and BP3. Many key interactions between these two proteins are conserved in the human orthologs CPSF160 and WDR33 (Fig. 3B and fig. S7).

**Fig. 3:**
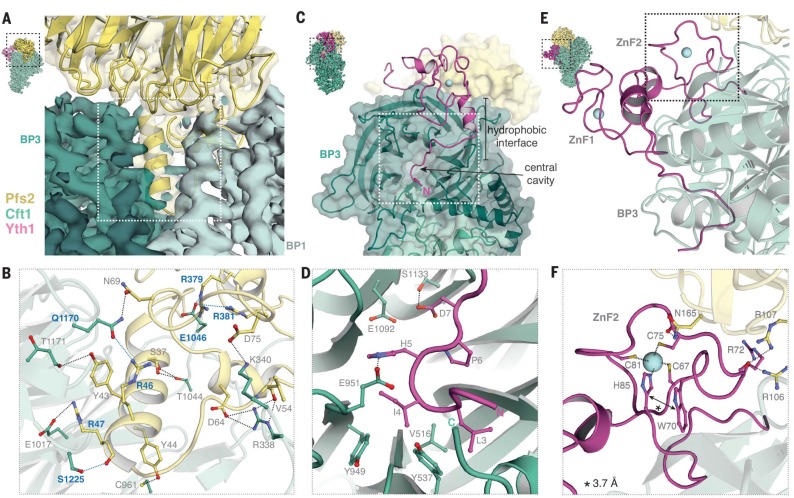
**Protein–protein interactions in the polymerase module. (A** and **B)** Details of the Cft1 (green)–Pfs2 (yellow) interaction. The cryo-EM map of Cft1 is shown (A). Selected interactions with residues conserved in human orthologs are labeled in blue (B). (**C** to **F**) Details of Yth1 (magenta) interaction with Pfs2 and Cft1. A surface representation of the Cft1 model is shown (C). Selected electrostatic and hydrophobic interactions are depicted (D). Zinc ions are in cyan (E). ZnF2 is stabilized by pi stacking between Yth1-H85 and -W70, as well as several hydrogen-bonding interactions between side chains of Pfs2 and backbone atoms of Yth1 (F). Single-letter abbreviations for the amino acid residues are as follows: C, Cys; D, Asp; E, Glu; H, His; I, Ile; K, Lys; L, Leu; N, Asn; P, Pro; Q, Gln; R, Arg; S, Ser; T, Thr; V, Val; W, Trp; and Y, Tyr.

Yth1 is anchored onto the complex by an extended N-terminal segment that binds in the central cavity of Cft1-BP3 and continues across a hydrophobic external face (Fig. 3, C and D). Next, two of the five Cys-Cys-Cys-His (CCCH) zinc fingers pack into the interface between Cft1 and Pfs2 (Fig. 3, E and F).

We performed cross-linking mass spectrometry to validate our structural model and to determine where Pap1 and Fip1 bind. Inter- and intramolecular cross-links agree with our atomic models, and the crystal structure of Pap1 (fig. S8 and table S4) (*20*). Fip1 cross-links to the Cterminal part of Yth1 and the polymerase domain of Pap1 (fig. S8B). A previous crystal structure of Pap1 in complex with a peptide of Fip1 (*15*) revealed molecular details of their interaction, but Pap1 also cross-links to the C-terminal helical domain of Cft1, ZnF1 of Yth1, and the C-terminal region of Pfs2 (fig. S8). Together, these data suggest that the flexible C-terminal half of Yth1 binds the intrinsically disordered protein Fip1, which in turn flexibly tethers Pap1 to the complex, allowing conformational freedom to add long poly(A) tails onto diverse RNA substrates.

The cryo-EM structure of the Cft1–Pfs2–Yth1 complex of the polymerase module has a markedly similar architecture to the eukaryotic DDB1- DDB2 and SF3b complexes (*21**–**24*) (Fig.4,AtoC). DDB1-DDB2 recognizes ultraviolet-damaged DNA and acts as an adapter for a cullin-RING E3 ubiquitin ligase to trigger nucleotide excision repair. SF3b is a multiprotein assembly containing the Rse1/SF3b130 scaffold protein, which forms part of the U2snRNP complex essential for pre-mRNA splicing and branch site recognition.

**Fig. 4:**
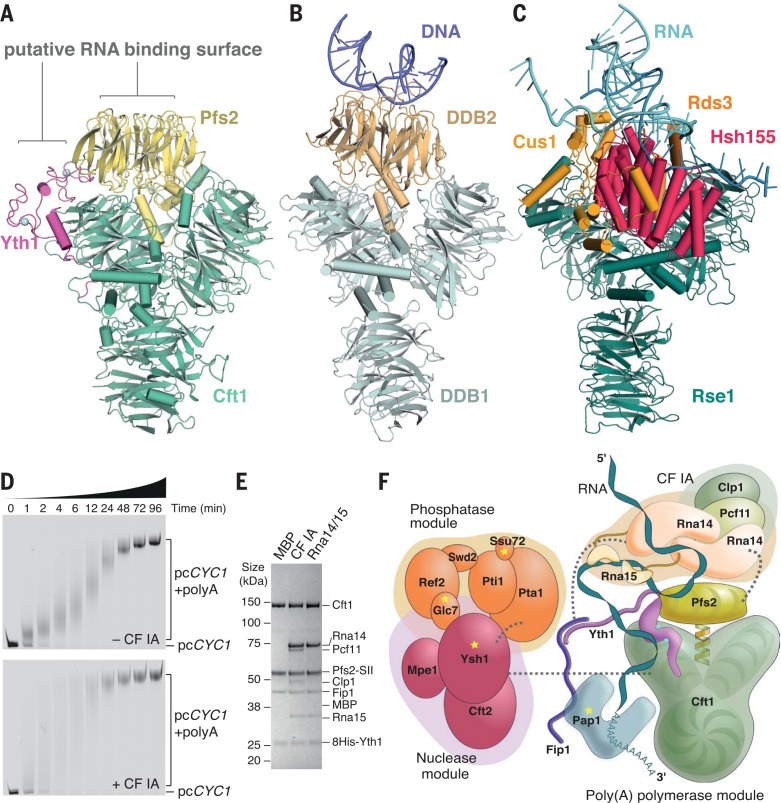
**The polymerase module acts as a hub to bring together Pap1, RNA, and accessory factors.** (**A** to **C**) The Cft1–Pfs2–Yth1 complex of the polymerase module (A) is structurally similar to the DDB1– DDB2 DNA repair [PDB: 3ei3 (B)] and SF3b splicing [PDB: 5gm6 (C)] complexes. **(D)** Polyadenylation of a fluorescently labeled 42-nucleotide precleaved (pc) CYC1 RNA by the polymerase module (with and without CF IA) analyzed by 15% denaturing urea PAGE. **(E)** Coomassie-stained SDS-PAGE of pulldown experiment showing immobilized polymerasemodule after incubation with purifiedmaltose-binding protein (MBP), CF IA, or Rna14–Rna15. **(F)** Model for the 3ʹ-end processing machinery obtained by combining data from nanoESI-MS, cryo-EM, cross-linking–mass spectrometry, and in vitro pull-down assays.Yellow stars denote enzymes.

DDB1 and Rse1 both contain three β propellers followed by a C-terminal a-helical domain, with the same fold as Cft1, and the three proteins show weak sequence homology (~15% sequence identity). Thus, all three complexes use similar scaffold proteins (DDB1, Rse1, or Cft1) to assemble a rigid and structurally stable complex. Their interaction partners (DDB2, Hsh155/Rds3, or Pfs2) bind in the same cavity between BP1 and BP3, with a similar binding mode that involves a helices, but the exact interaction mechanism is not conserved (Fig. 4, A to C). Like DDB1-DDB2 and SF3b, Cft1 may bind additional subunits through its β propellers.

DDB1-DDB2 and SF3b directly bind nucleic acid. The polymerase module also binds RNA in a gel-shift assay (fig. S9A). The surface of Pfs2 equivalent to the DDB2 DNA binding site contains a cluster of conserved lysines, arginines, and aromatic residues that could form an RNA interaction surface (fig. S9, β to D). This Pfs2 surface lies adjacent to the RNA-binding ZnF2 of Yth1 (*25**–**27*) and together, they might constitute a composite RNA-binding platform (Fig. 4A) that is disrupted in viral infections (fig. S9E).

Intact CPF requires the accessory cleavage factors (CF) IA and IB for efficient and specific polyadenylation (*28**–**30*). Addition of recombinant CF IA (but not CF IB) stimulates the polyadenylation activity of the polymerase module and intact CPF (Fig. 4Dand fig. S10, A to C). CF IA has no effect on isolated Pap1 (fig. S10D), underscoring the functional importance of the other subunits.

CF IA is composed of four different protein subunits–a heterotetramer of Rna14–Rna15 and a heterodimer of Pcf11-Clp1. Rna14–Rna15 was sufficient to stimulate polyadenylation (fig. S10E). Moreover, the CF IA complex, and specifically the Rna14–Rna15 subcomplex, binds to the polymerase module in pull-down assays (Fig. 4E and fig. S10F).

The arrangement of CPF, in which its enzymatic activities are segregated into three modules, suggests that coupling between the enzymes is not through intimate, stable contacts and may be dynamic. CF IA likely stimulates polyadenylation by contributing additional RNA binding sites. Together, Pfs2, Yth1, and CF IA could stably bring specific RNA sequences to the complex. This would allow Pap1, which is flexibly tethered to the complex by the intrinsically disordered protein Fip1, to access a variety of different RNA substrates for efficient and controlled polyadenylation. Thus, the polymerase module acts as a hub to bring together Pap1, substrate RNA, and CF IA (Fig. 4F). Moreover, by tethering these components together with the nuclease and phosphatase modules of CPF, it would facilitate accurate 3′-end processing, and coordination with transcription.

## Supplementary Material

Architecture of eukaryotic mRNA 3′-end processing machinery

Architecture of eukaryotic mRNA 3′-end processing machinery

Architecture of eukaryotic mRNA 3′-end processing machinery

## SUPPLEMENTARY MATERIALS

www.sciencemag.org/content/358/6366/1056/suppl/DC1

Tables S1 to S4

References (*31–76*)
