# Supplementary material for: Architecture of eukaryotic mRNA 3′-end processing machinery
Source: Science. 2017 Oct 26;358(6366):1056–9. doi: 10.1126/science.aao6535 (PMC5788269; doi:10.1126/science.aao6535)
Supplement: Architecture of eukaryotic mRNA 3′-end processing machinery [file Science-358-1056-s1.pdf]

www.sciencemag.org/cgi/content/full/science.aao6535/DC1

Supplementary Materials for

Architecture of eukaryotic mRNA 3′-end processing machinery

Ana Casañal, Ananthanarayanan Kumar, Chris H. Hill, Ashley D. Easter, Paul Emsley, Gianluca Degliesposti, Yuliya Gordiyenko, Balaji Santhanam, Jana Wolf, Katrin Wiederhold, Gillian L. Dornan, Mark Skehel, Carol V. Robinson, Lori A. Passmore*

*Corresponding author. Email: passmore@mrc-lmb.cam.ac.uk

Published 26 October 2017 on *Science* First Release
DOI: 10.1126/science.aao6535

This PDF file includes:

Materials and Methods
Figs. S1 to S10
Tables S1 to S4
References
Caption for Movie S1
Caption for Additonal Data S1

Other Supplementary Materials for this manuscript include the following:
(available at www.sciencemag.org/cgi/content/full/science.aao6535/DC1)

Movie S1
Additional Data S1

Materials and Methods

Native CPF purification and activity assays

Endogenous CPF was purified from a yeast strain where *PAP1*, *PTA1*, *REF2*, *SYC1* or *MPE1* was modified to contain a TAPS tag as in described previously (*7*). CPF was further purified using a 1 ml Mono Q 5/50 GL column (GE Healthcare) equilibrated in buffer T (20 mM HEPES pH 7.9, 150 mM KCl, 0.5 mM $Mg(OAc)_2$ and 3 mM DTT) and eluted in a two-step gradient at 260 mM KCl and 400 mM KCl.

Activity assays were performed at 30 °C in 40 µl reaction volumes in assay buffer (5 mM HEPES pH 8.0, 75 mM KOAc, 2 mM $Mg(OAc)_2$, 2 % (v/v) PEG6000) with 1 mM DTT, 1 U RiboLock Inhibitor (ThermoScientific), and 100 nM *CYC1* RNA (*28*) (produced by in vitro transcription). 2 mM ATP was used for polyadenylation assays. Proteins were added at the following final concentrations: 50 nM CPF, 150 nM CF IA, 150 nM CF IB. Reactions were stopped by adding an aliquot to stop solution (130 mM EDTA, 5% (w/v) SDS, 12 mg/ml proteinase K made in assay buffer) and incubating at 37 °C for 30 min. Samples were run on a 6% Urea/TBE (Tris-borate-EDTA) gel (LifeTechnologies), stained with SyBr Green II and imaged on a ChemiDoc XRS+ (BioRad).

CF IA purification

The CF IA complex (Rna14, Rna15, Pcf11, and Clp1) was purified using a modified version of a previously-described method (*31*). Expression plasmids were kindly provided by Andrew Bohm (Tufts School of Medicine, Boston). BL21(pLysS) Rosetta cells were used for expression. 4 L pETDuet–His-Rna15–Rna14 cells in 2XTY were grown at 37 °C to an OD_{600} of 0.6 prior to induction (1 mM IPTG, 16 °C, overnight). Cells containing pRSFDuet–His-Pcf11–Clp1 were grown in baffled flasks at 37 °C in 500 ml media containing 0.5% glucose and 1 mM $MgSO_4$ to an OD_{600} of 2.0. Expression was induced by adding 500 ml of fresh 2XTY media containing IPTG (final 1 mM IPTG, 30 °C, 3 h).

For Rna14–Rna15 purification, cells were lysed in buffer A (50 mM HEPES pH 8.0, 250 mM NaCl, 30 mM imidazole, 5% w/v glycerol, 0.5 mM TCEP, 2 µg/ml DNase I (Sigma), complete EDTA-free protease inhibitors (Roche)) by sonication at 4 °C. After clarification (39,000*g*, 30 min), lysate was filtered (0.45 µm) and loaded onto a 5 ml HisTrap HP column (GE) pre-equilibrated with buffer B (20 mM HEPES pH 8.0, 250 mM NaCl, 0.5 mM TCEP). Proteins were eluted with a 10 column volume (CV) gradient up to 100% buffer C (20 mM HEPES pH 8.0, 250 mM NaCl, 500 mM imidazole, 0.5 mM TCEP). Fractions containing Rna14–Rna15 were pooled and concentrated using a 30 kDa Amicon® Ultra centrifugal filter (Millipore) prior to size exclusion chromatography with a Superdex 200 16/600 column (GE) equilibrated in buffer B. Peak fractions were pooled and concentrated to 100 µM. Aliquots were flash-frozen in liquid nitrogen and stored at -80 °C.

For the His-Pcf11–Clp1 purification, cells were lysed in buffer D (50 mM Tris pH 8.0, 250 mM NaCl, 5% w/v glycerol, 1 mM TCEP, 2 µg/ml DNase I, 2 µg/ml RNase A, 1 tablet of EDTA-free protease inhibitor tablets per 50 ml) by sonication. Lysates were clarified and filtered as above before loading onto a 5 ml HisTrap FF crude column (GE)

pre-equilibrated with buffer E (20 mM Tris pH 8.0, 250 mM NaCl, 1 mM TCEP). The column was washed with 20 mM Tris pH 8.0, 20 mM imidazole, 250 mM NaCl, 1 mM TCEP for 10 CV prior to gradient elution (10 CV) up to 100% buffer F (20 mM Tris pH 8.0, 250 mM NaCl, 500 mM imidazole, 0.5 mM TCEP).

30 mg of purified Rna14–Rna15 and Pcf11–Clp1 were mixed 1:1 (w/w) and incubated on ice overnight. The complex mixture was diluted to 100 mM NaCl, clarified by centrifugation (8000*g*, 10 min), filtered (0.45 μm) and loaded onto a 5 ml HiTrap Q HP column (GE). The column was washed with 20 mM Tris pH 8.0, 100 mM NaCl, 1 mM TCEP for 5 CV prior to gradient elution (6 CV) up to 100% buffer G (20 mM Tris pH 8.0, 1 M NaCl, 1 mM TCEP). The complex was then further purified by size exclusion chromatography using a Superdex 200 26/600 column (GE) equilibrated in buffer E. CF IA was concentrated to 24 μM as above, flash-frozen in liquid nitrogen and stored at -80 °C.

CF IB purification

The *HRP1* gene was cloned into a pOPINB vector (Oxford Protein Production Facility) and verified by sequencing, yielding N-terminally His-tagged Hrp1. Protein was expressed in 3 L BL21(DE3) Rosetta pLysS cells grown at 37 °C to an OD_{600nm} of 0.6 prior to induction (1 mM IPTG, 37 °C, 3 h).

The harvested cells were lysed as above, in buffer H (50 mM HEPES pH 8.0, 300 mM NaCl, 20 mM imidazole, 0.5 mM TCEP) with 10% w/v glycerol, 2 μg/ml DNase I, 2 μg/ml RNase A, EDTA-free protease inhibitors. Clarified lysate was mixed with 1 ml bed volume of Ni-NTA beads pre-equilibrated in buffer H and incubated for 75 min at 4 °C. Beads were washed twice with 50 ml buffer H and once with 50 ml buffer I (50 mM HEPES pH 8.0, 300 mM NaCl, 30 mM imidazole, 0.5 mM TCEP). Beads were transferred to a gravity column before elution with buffer J (50 mM HEPES pH 8.0, 150 mM NaCl, 300 mM imidazole, 0.5 mM TCEP). Protein was then loaded onto a 1 ml HiTrap Heparin column (GE) equilibrated in 20 mM HEPES pH 8.0, 100 mM NaCl, 0.5 mM TCEP prior to gradient elution (20 CV) up to 100% buffer K (20 mM HEPES pH 8.0, 1 M NaCl, 0.5 mM TCEP). Fractions containing Hrp1 were pooled and concentrated with a 10 kDa Amicon® Ultra centrifugal unit prior to size exclusion chromatography with a Superdex 200 10/300 column (GE) equilibrated in buffer B. Hrp1 was then concentrated as above to 104 μM, flash-frozen in liquid nitrogen and stored at -80 °C.

Pap1 purification

The *PAP1* gene was cloned into a pET-28a(+) vector, fusing it to an N-terminal His-tag. 2 L cultures of BL21(DE3) Star cells containing pET-28a–His-Pap1 were grown at 37 °C to an $OD_{600\ nm}$ of 0.6 prior to induction (1 mM IPTG, 16 °C, overnight). Sonication was performed in buffer L (50 mM HEPES pH 8.0, 1 M NaCl, 20 mM imidazole, 5% w/v glycerol, 1 mM TCEP, 2 μg/ml DNase I, 2 μg/ml RNase A, EDTA-free protease inhibitors). Clarified lysate was mixed with 1 ml bed volume of Ni-NTA resin pre-equilibrated with buffer M (50 mM HEPES pH 8.0, 500 mM NaCl, 20 mM imidazole, 1 mM TCEP) and incubated for 120 min at 4 °C. The resin was then washed twice with 50 ml buffer M. Beads were transferred to a gravity column before elution with buffer N (50 mM HEPES pH 8.0, 500 mM NaCl, 300 mM imidazole). Fractions containing Pap1 were exchanged into buffer O (50 mM HEPES pH 8.0, 100 mM NaCl, 0.5 mM TCEP), and

loaded onto a 1 ml HiTrap Heparin HP (GE). Proteins were eluted over a 30 CV gradient up to 100% buffer P (50 mM HEPES pH 8.0, 1 M NaCl, 0.5 mM TCEP). Pap1 was further purified by size exclusion chromatography using a Superdex 200 10/300 column equilibrated in buffer Q (50 mM HEPES pH 8.0, 150 mM NaCl, 1 mM TCEP). Pap1 was then concentrated to 36 µM, flash-frozen in liquid nitrogen and stored at -80 °C.

nanoESI-MS

We analyzed CPF complex from multiple strains, each containing a TAP tag on a different subunit, to differentiate between proteins with similar molecular weights. For native nanoESI-MS, the protein complex needs to be in a volatile buffer, most commonly ammonium acetate. We tried three methods for buffer exchange. First, we used concentration and drop-dialysis into 500 mM ammonium acetate after StrepTactin elution of CPF. Second, after washing with 500 mM ammonium acetate, we eluted CPF from the StrepTactin resin directly into 500 mM ammonium acetate with 5 mM desthiobiotin. Desthiobiotin was removed by concentration of the protein using 50 kDa Amicon® Ultra centrifugal filters followed by dilution in 500 mM ammonium acetate, a total of five times. Third, we exchanged the buffer using size exclusion chromatography as follows: CPF in normal elution buffer was concentrated from ~1.5 ml to 50 µl using 50 kDa Amicon® Ultra centrifugal filters and injected onto a Superose 6 PC 3.2/30 column (GE) pre-equilibrated in 350 mM ammonium acetate pH 8.2 (at 4 °C). The column was run at 1.0 ml/min on an Äkta micro FPLC system (GE). 50 µl fractions were collected. Protein-containing fractions were pooled and concentrated to 30–60 µl (~1 mg/ml). This final method for buffer exchange worked best but results from all methods are included here.

MS and tandem MS (MS/MS) spectra were acquired on a high mass Q-TOF-type instrument adapted for a QSTAR XL platform (MDS Sciex) (*32*) using in-house prepared gold-coated glass capillaries (*11*, *33*). Spectra were calibrated externally using a solution of caesium iodide (100 mg/ml).

2.5 µl of each sample was taken up in gold-coated glass capillaries, attached to the nanoflow backing pressure line, connected to the high tension and aligned with the sampling cone to ensure good sample spray (determined by ensuring a high and stable total ion current (TIC)). Instrument parameters were optimized using starting parameters of: capillary voltage 1.3 kV, declustering potential (cone voltage) 100–120 V, focusing potential 200 V, collision energy up to 200 V, micro-channel plate detector (MCP) 2350 V (*34-36*). For MS/MS the target m/z range was selected in the second quadrupole and subjected to acceleration in the collision cell, using argon gas at maximum pressure (4 MPa). In both MS and MS/MS modes, the m/z range collected was optimized to include ions of interest and suppress noise from adducts and low molecular weight contaminants. A range of 1,000–24,000 m/z was used initially, narrowing down once the ionization of the sample was known. Most MS data were collected between 1,000 and 16,000 m/z, with all spectra in positive-ion mode.

Spectra were acquired using *MassLynx MS* software (Waters) and processed following previous methods (*37*) but without the *Massign* software package. Each spectrum was smoothed to remove high-frequency noise, peak m/z values assigned and charge series identified via least-squares fitting. Peak series were verified visually by the presence of expected p_{z-1} and p_{z+2} peaks. The standard deviation in fitting the identified peaks to the charge series is given as the ± error in the measured mass. This is the error in

the fit and not the error in the mass measurement, which is likely an order of magnitude higher due to solvation/adduct effects, heterogeneous post-translational modifications, etc. Hence, the error gives a rough measure of the accuracy of peak assignment, which is impacted by the broadness, symmetry and signal:noise of each peak.

The mass of each protein subunit was calculated from amino acid composition using *ProtParam* (http://web.expasy.org/protparam/), with protein identity confirmed using peptide fingerprinting. To understand the connectivity within CPF and APT, raw data were combined computationally into interaction networks. Using the *SUMMIT* program in network explorer mode (*38*), possibilities for each experimental mass were calculated using a ± 1000 ppm window (*e.g.* a tolerance of ± 500 Da for a 500 kDa complex). After initial clustering with the *SUMMIT* program, redundant connections were removed manually.

Some direct binary interactions could not be confirmed – these are indicated by grey lines on Fig. 1C. Specifically, for Pti1 binding to Pta1–Syc1, only the trimeric complex was detected and for Fip1 binding to Cft1–Pfs2–Yth1 only the tetrameric complex was detected. An isolated binary complex of Cft1–Pap1 was not detected, but this interaction may be direct because both Cft1–Yth1–Pap1 and Cft1–Pfs2–Pap1 complexes were observed.

<u>Overexpression and pulldown in yeast</u>

CFT1, *CFT2*, *YSH1* and *PTA1* were cloned into the pRS424 or pRS426 vectors designed for overexpression of proteins in *S. cerevisiae* (*39*). Pairs of proteins were co-overexpressed, with one of them containing a StrepII-tag, in BCY123 yeast cells. 5 ml of YM4 (+Trp or Ura, if necessary) supplemented with 2 % raffinose were inoculated and incubated for 16 h at 30 °C and 200 rpm for their use as starter cultures. Next, 50 ml cultures of YM4 (+Trp or Ura, if necessary) supplemented with 1% raffinose were inoculated with the starter culture to an $OD_{600\ nm}$ of 0.2, and grown at 30 °C and 200 rpm until an $OD_{600\ nm}$ of ~1 was reached. Protein expression was induced by addition of galactose to a final concentration of 2% and cells incubated for 14–16 h at 30 °C and 200 rpm. After harvesting, cells were washed in milliQ water, pelleted, the supernatant removed, and the washed pellets flash frozen in liquid nitrogen for storage at -80 °C.

Cells were resuspended in 2 ml buffer R (50 mM HEPES pH 8.0, 300 mM NaCl, 1% w/v glycerol, 2 mM β-ME, complete EDTA-free protease inhibitor tablets and 0.1 µg/ml DNaseI) and lysed by bead beating using glass beads for 10 min at 4 °C. Lysates were incubated with 50 µl StrepTactin Sepharose (GE Healthcare) for 1 h at 4 °C. Beads were washed with in buffer R (5 x 1 ml) and protein was eluted by incubation with 100 µl buffer R with 5 mM desthiobiotin for 45 min at 4 °C. Elution fractions were analyzed on 4–12% NuPage Novex Bis-Tris gels (Life Technologies). *S. cerevisiae* proteins acetyl CoA carboxylase (Acc1, 250 kDa) and pyruvate carboxylase (Pyc1 and Pyc2, 130 kDa) bind non-specifically to StrepTactin Sepharose and are labelled with asterisks on all gel pictures.

<u>Cloning, expression and purification of recombinant polymerase module</u>

Codon-optimized genes were synthesized by Geneart and cloned into pACEBac1 (Cft1, Pfs2-Strep, Yth1) or pIDC (Pap1, Fip1) to generate expression constructs flanked by a polyhedrin promoter and SV40 terminator. Multi-gene constructs were then

assembled as described in the MultiBac protocol (*40*) using I-CeuI/PI-SceI and BstXI restriction sites to yield pACEBac1–Cft1–Pfs2-StrepII–8His-Yth1 and pIDC-Fip1–Pap1. These two plasmids were then combined by Cre-Lox recombination and the resultant five-gene construct transformed into DH10EmbacY cells. Bacmid DNA was isolated and transfected into *Sf*9 cells as described previously (*41*). The supernatant (P1 virus) from the transfection wells was harvested 72 h post transfection, supplemented with 50% FBS and stored at 4 °C. The P1 virus was then amplified by further infection of *Sf*9 cells at $2x10^6$/ml (27 °C, 140 rpm, 72 h) to yield a high-titre P2 virus. Large-scale infections for protein expression were carried out in 500 ml cultures of *Sf*9 cells at $2x10^6$/ml with 1:100 (v/v) P2 virus (27 °C, 140 rpm, 72 h). Cells were harvested post infection by centrifugation (3000*g*, 10 min), washed in PBS, flash-frozen in liquid nitrogen and stored at -80 °C.

Cell pellets from 2 l *Sf*9 cells were resuspended in buffer S (50 mM HEPES pH 7.9, 300 mM NaCl, 1 mM TCEP) with 50 µg/ml RNAse, 50 µg/ml DNAse and EDTA-free protease inhibitors and lysed by sonication as above. Lysate was cleared by ultracentrifugation (100,000*g*, 30 min) prior to incubation (4 °C, 2 h) with 2 ml of StrepTactin Sepharose HP resin pre-equilibrated with buffer S. Beads were loaded onto a gravity column and washed with 150 ml buffer S prior to elution with buffer S supplemented with 6 mM of desthiobiotin. The eluate was diluted with 50 mM HEPES pH 7.9 to reduce the salt concentration to 150 mM NaCl. The sample was then loaded onto a 1 ml Mono Q 5/50 GL column (GE Healthcare) and eluted over a 45 CV gradient from 300–450 mM NaCl. This separated polymerase modules with and without Pap1. The four-subunit complex (without Pap1) was the most abundant species. Individual peak fractions from the anion exchange step were concentrated using a 50 kDa Amicon® Ultra centrifugal filter and were subsequently purified by size exclusion chromatography using a Superose 6 3.2/300 Increase column (GE Healthcare) pre-equilibrated with buffer S. Purified polymerase module was either used immediately for making cryo-EM grids, or was concentrated as above (10–20 µM), flash frozen in liquid nitrogen and stored at -80 °C for further biochemical assays.

Sample preparation and cryo-EM data collection

Endogenous CPF purified by MonoQ was crosslinked with 2 mM BS3 (Sigma) for 5 min at 30 °C, and the reaction quenched with 50 mM ammonium bicarbonate. Cryo samples of endogenous CPF were prepared on ultrastable gold supports (UltraAuFoil R1.2/1.3) (*42*). Grids were glow-discharged for 30 s before deposition of 3 µl sample (~0.1 mg ml^{-1}), blotted for 8 s, and vitrified by plunging into liquid ethane with a custom-made manual plunger at 4 °C. Micrographs were collected on a FEI Titan Krios microscope operated at 300 keV and equipped with a Falcon II detector. 795 micrographs were collected at MRC LMB (Krios 1) with a calibrated pixel size of 1.77 Å. Movies with a total electron dose of ~40 $e^-/Å^2$ were recorded in integration mode over 2 s and a defocus range of 2–4.5 µm.

To perform cryo-EM studies of the poly(A) polymerase module, freshly-prepared sample was vitrified on cryo-EM grids. Aliquots of 3 µl of sample were applied onto glow-discharged UltraAuFoil R1.2/1.3 gold supports, blotted for 3 s and plunge-frozen in liquid ethane using an FEI Vitrobot MKIII, at 100% humidity and 4 °C. The same procedure described above to prepare cryo-EM grids of endogenous CPF was used to

vitrify the sample on Quantifoil R1.2/1.3 grids coated with graphene oxide (*43*, *44*). Initial studies showed that the five-subunit polymerase module (with Pap1) was heterogeneous compared to the four-subunit complex (without Pap1). Thus, the latter specimen was used for data collection. Micrographs were collected on FEI Titan Krios microscopes operated at 300 keV and equipped with a K2 detector and a Gatan Imaging Filter (GIF) with a slit width of 20 eV. 3,413 micrographs were collected at MRC LMB (Krios 2) with a calibrated pixel size of 1.4 Å, and 814 micrographs were collected Diamond Light Source (DLS) electron Bio-Imaging Centre (eBIC) with a calibrated pixel size of 1.27 Å. Movies with a total electron dose of ~45 $e^{-}/Å^{2}$ were recorded in super-resolution mode over 16 s (20 frames, 0.8 s per frame) and a defocus range of 1.5–3.5 μm.

Image processing

For cryo-EM analysis of cross-linked CPF, frames were aligned and averaged with *MotionCorr* (*45*). Contrast transfer function (CTF) parameters were calculated with *Gctf* (*46*). All subsequent steps were performed in *RELION-2* (*47*, *48*). Initially, a subset of micrographs was used to manually pick ~1,000 particles for initial reference-free 2D classification. The resulting 2D classes were used for automated particle-picking in *RELION-2*. Sorting of particle images was performed by 2D and 3D classification. The *ab initio* 3D model employed for 3D refinements was obtained using the sorted 2D classes as input in *SIMPLE-PRIME* (*49*). 41,577 particles contributed to the final map with an overall resolution of ~12 Å (fig. S3).

Datasets from different microscopes were processed separately to obtain independent high-resolution (~4 Å) 3D reconstructions of the poly(A) polymerase module as described above with the following variations. Frames were aligned, averaged and downscaled with *MotionCor2* (*50*). The 3D model employed for 3D refinements was created by low-pass filtering (40 Å) the 3D reconstruction obtained from the native cross-linked CPF sample. The particles extracted from the eBIC 3D reconstruction were rescaled in *RELION-2* to 1.4 Å pixel size to obtain new 2D classes. Particle-based movement correction (particle polishing) and per-frame B-factor weighting were performed independently for each dataset in *RELION-2*. The resulting shiny particles were then combined, yielding a total of 460,167 particles, which were subjected to 3D classification (without alignments and with no resolution limit) to separate structural heterogeneity and discard remaining bad particles (fig. S4). The subset of 77,917 (17% of total particles) contributed to the final map with an overall resolution of 3.5 Å (fig. S4 and S5). The resolution estimation reported is based on the gold standard Fourier shell correlation (FSC) at 0.143, and the calculated FSC is derived from comparisons between reconstructions from two independently refined half-sets. Local resolution was estimated using *RELION-2* (fig. S5).

Model building

We used homology models of the domains of Cft1 and Pfs2 obtained from *Phyre2* (*51*) and based on the crystal structure of the DDB1-DDB2 complex (*22*) (PDB 3EI3), in a hybrid approach to build the full atomic model of the polymerase module. First, each beta propeller domain was fitted and initially refined using jiggle fit and morphing with *Coot* (*52*, *53*). On initial inspection of the map, although the backbone was traced *de novo*

with confidence, the sequence and identity of the Yth1 component was not apparent. However, the characteristic shape of the unidentified density hinted at the possibility of a zinc finger. To test this hypothesis we performed molecular replacement with the crystal structure of CPSF30 (*19*) (PDB 2RHK). This provided a solution sufficiently close for us to unambiguously identify the unassigned density as that of Yth1. The model was refined with *Refmac v.5.8* (*54*) and *phenix.refine* (*55*) and validated with *MolProbity* (*56*). Atomic B factors given in the pdb file are from the *Refmac* refinement. Maps were analyzed and visualized using *Chimera* (*57*) and figures were prepared using *PyMOL* (http://www.pymol.org). Electrostatic potentials were calculated by solving the Poisson–Boltzmann equation with the APBS plugin in *PyMOL* using the default settings (pH 7).

Polyadenylation, pulldown and gel shift assays with polymerase module

A 42 nt pre-cleaved *CYC1* (pc-*CYC1*) mRNA (*58*), chemically synthesized with a 5′ 6-FAM fluorophore (IDT) was used as a substrate in the polyadenylation assays carried out with the recombinant complex. Polyadenylation assays were performed in 10 mM HEPES pH 7.9, 150 mM KOAc, 2 mM $Mg(OAc)_2$, 0.05 mM EDTA, 2% PEG (v/v) with 1 mM DTT and at 30 °C. Purified protein complexes were diluted to a concentration of either 500 nM (polymerase module, Pap1 or CPF) or 4 µM (CF IA or Rna14–Rna15) in 50 mM HEPES pH 7.9, 150 mM NaCl, 1 mM TCEP before being used in the assays. A master mix containing 400 nM pc-*CYC1* was incubated for 5 minutes with 50 nM protein (polymerase module, Pap1 or CPF) and the reaction was started by addition of ATP at a final concentration of 2 mM. For assays with CF IA, the master mix containing 400 nM pc-*CYC1* was pre-incubated with 400 nM CF IA for 5 minutes before the addition of proteins (polymerase module or CPF).

For assays comparing the activity of the polymerase module and CPF, the purified protein complexes were first resolved by 4–12% SDS-PAGE (Invitrogen) and visualized by Coomassie staining. The concentration of polymerase module to be used in the polyadenylation assays was determined by normalizing for the SDS-PAGE band intensity of Pap1 in each of the complexes.

For assays with CF IB, the master mix containing 300 nM pc-*CYC1* was pre-incubated with 300 nM CF IB for 5 minutes before the addition of polymerase module.

Samples of 10 µl were taken at appropriate time-points and mixed with denaturing formamide RNA loading dye. Products were analyzed by running on a urea-polyacrylamide gel (15% [w/v] 19:1 acrylamide: bisacrylamide, 7 M urea, 1xTBE) for 30 minutes at 400 V followed by imaging on a Typhoon FLA-7000 laser scanner (GE Healthcare).

For protein–protein pulldown experiments, 20 µl of Strep-Tactin XT coated magnetic beads (IBA) were equilibrated with 100 µl of buffer S150 (50 mM HEPES pH 7.9, 150 mM NaCl, 1 mM TCEP), mixed with 20 µl of 1.5 µM purified polymerase module (the bait), and incubated for 1 hour at 4 °C in a microplate shaker. For strep control pulldowns, 20 µl of buffer S150 was used as bait. After incubation, the supernatant was removed and the beads were washed twice with 100 µl of buffer S150 with 0.01% Tween-20 (Sigma). 20 µl of 1.5 µM purified prey (either MBP as a control, CF IA, Rna14–Rna15 or Pap1) was mixed with 20 µl of the bait-loaded beads and incubated for 1 hour at 4 °C in a microplate shaker. The beads were then washed 5 times with 200 µl of buffer S150 with 0.01% Tween-20. Once the excess wash buffer was

removed, the beads were resuspended in 30 μl of 4X LDS sample buffer (Invitrogen). The protein complexes were resolved by 4–12% SDS-PAGE (Invitrogen).

For EMSA assays, 8 μl of the purified four-subunit polymerase module (at final concentrations 125 nM, 250 nM, 500 nM, 1 μM and 2 μM) was incubated with 1 μl of 500 nM pc-*CYC1* RNA (final concentration 50 nM) and 1 μl of 10X orange loading dye (0.4% w/v orange G, 1 mM EDTA pH 8, 50% v/v glycerol). The protein dilution series was carried out in buffer S150 containing 1 U RiboLock Inhibitor (ThermoScientific). The protein RNA mixture was incubated on ice for 20 minutes before running the products on a native-polyacrylamide gel (6% [w/v] 19:1 acrylamide: bisacrylamide, 1xTBE) for 40 minutes at 100 V. The gel was imaged on a Typhoon FLA-7000 laser scanner (GE Healthcare).

Crosslinking-MS

Cross-linking reactions were performed using purified polymerase module (with or without Pap1) at a concentration of 1 mg ml^{-1} in 50 mM Hepes pH 7.9. Homobifunctional, isotopically-coded N-HydroxySuccinimide (NHS) ester BS3 (H12/D12), purchased from Creative Molecules (Canada), was used at a concentration of 600 μM to target lysine residues. The reactions were incubated for 45 min at 37 °C and quenched by adding NH_4HCO_3 to a final concentration of 50 mM and incubating for further 15 min.

Cross-linked samples were freeze-dried and resuspended in 50 mM NH_4HCO_3 to a final protein concentration of 1 mg ml^{-1}, reduced with 10 mM DTT and alkylated with 50 mM iodoacetamide. Next, trypsin was added at an enzyme:substrate ratio of 1:20 and digestion was carried out overnight at 37 °C. After digestion, the samples were acidified with formic acid to a final concentration of 2% v/v.

Digests were then fractionated by peptide-level size exclusion chromatography using a Superdex Peptide 3.2/300 (GE Healthcare) with a 30% v/v Acetonitrile 0.1% v/v TFA mobile phase at a flow rate of 50 μl min^{-1}. Fractions were collected every 2 min from 1.0–1.7 ml elution volume. Fractions were freeze-dried and resuspended in 2% v/v acetonitrile and 2% v/v formic acid.

The digests were analyzed by nano-scale capillary LC-MS/MS using an Ultimate U3000 HPLC (ThermoScientific Dionex, San Jose, USA) to deliver a flow of approximately 300 nL min^{-1}. A C18 Acclaim PepMap100 5 μm, 100 μm x 20 mm nanoViper (ThermoScientific Dionex), trapped the peptides prior to separation on a C18 Acclaim PepMap100 3 μm, 75 μm x 250 mm nanoViper (ThermoScientific Dionex). Peptides were eluted with a gradient of acetonitrile. The analytical column outlet was directly interfaced via a nano-flow electrospray ionization source, with a hybrid dual pressure linear ion trap mass spectrometer (Orbitrap Velos, ThermoScientific). Data dependent analysis was carried out, using a resolution of 30,000 for the full MS spectrum, followed by ten MS/MS spectra in the linear ion trap. MS spectra were collected over a m/z range of 300–2000. MS/MS scans were collected using threshold energy of 35 for collision-induced dissociation.

Thermo Xcalibur .raw files were converted into the open mzXML format through *MSConvert* (Proteowizard) with a 32-bit precision. mzXML files were directly used as input for *xQuest* searches on a local *xQuest* installation (*59*). The selection of cross-link precursors MS/MS data was based on the following criteria: a mass difference among the

heavy and the light cross-linker of: 12.07532 Da; precursor charge ranging from 3+ to 8+; maximum retention time difference 2.5 min.

Searches were performed against an ad-hoc database containing all the sequences of the complex together with their reverse used as decoy database. The following parameters were set for *xQuest* searches: maximum number of missed cleavages (excluding the cross-linking site) 3; targeted residues K, S, Y; peptide length 4–50 amino acids; fixed modifications carbamidomethyl-Cys (mass shift 57.02146 Da); mass shift of the light cross-linker 138.06808 Da; mass shift of mono-links 156.0786 and 155.0964 Da; MS1 tolerance 10 ppm, MS2 tolerance 0.2 Da for common ions and 0.3 for cross-link ions; search in enumeration mode (exhaustive search). Search results were filtered according to the following criteria: MS1 mass tolerance window –3 to 7 ppm. Finally, each MS/MS spectra was manually inspected and validated.

The shortlisted cross-linked peptide were checked on the structural model of the polymerase module reported and the previously determined Pap1 model (*60*) (PDB 2Q66). A distance cut-off of 27 Å was applied to validate the cross-links. In case of the targeted residues missing in the structure, but in close proximity to the terminal residues, the distance with the N- or C-terminal was evaluated. The remaining cross-links with one or both the residues in unmodeled regions were ranked according *xQuest* score, pair of residues involved in the cross-link: K-K highest priority, K-Y/S medium priority and Y/S-Y/S low priority.

<u>Bioinformatics analyses</u>

The orthologs of Cft1 and Pfs2 were identified using a combination of *PSI-BLAST* and *JackHMMER* (*61*) sequence searches against non-redundant and Uniprot databases respectively, using an e-value cut-off of 0.001. Multiple sequence alignment of the detected orthologs was constructed using *MSAProbs* (*62*). The alignments were refined based on *BLAST* and *JackHMMER* sequence profiles and *DSSP* (*63*, *64*) secondary structure assignment of reference protein Cft1 and Pfs2 3D structures. Interacting residues of Cft1 and Pfs2 were identified based on any of the following criteria: 1) *PISA* (*65*) for hydrogen bonds and salt bridges, 2) custom written *PERL* scripts to decipher non-covalent contacts based on van-der-Waal contact criteria (*66*), and 3) using Cα-Cα distance being larger than Cβ-Cβ distances, between residues that we identified as having hydrophobic contact with the Cα-Cα distance within 5 Å. Equivalent residue positions between yeast and human for both Cft1 and Pfs2 orthologs were identified for the above-determined interaction mediating residues based on their respective multiple sequence alignments. The interaction mediating equivalent residues between yeast and human were evaluated for sequence conservation based on BLOSUM62 similarity scores. We deemed any positive score between equivalent residues as some level of sequence conservation. Overall residue interactions between Cft1 and Pfs2 have been represented as a network using *Cytoscape* (*67*, *68*). Disorder predictions of proteins were carried out using *PrDOS* (*69*).

Author contributions:

A.C. and A.K. imaged complexes using cryo-EM, determined and interpreted the structure, and built atomic models; A.C., A.K. and K.W. performed activity assays; A.C., A.K., C.H.H. and A.D.E. purified protein complexes; A.D.E., Y.G. and C.V.R.

performed nanoESI-MS; P.E. assisted with model building; B.S. performed bioinformatics analyses; G.D. and M.S. performed cross-linking mass spectrometry; A.K. and J.W. performed pulldowns; A.C., C.H.H, and G.L.D. made constructs for baculovirus expression; A.C., A.K., C.H.H., A.D.E. and L.A.P. designed experiments and interpreted data throughout the project; L.A.P. conceived and supervised the project; A.C., A.K, and L.A.P. wrote the manuscript.

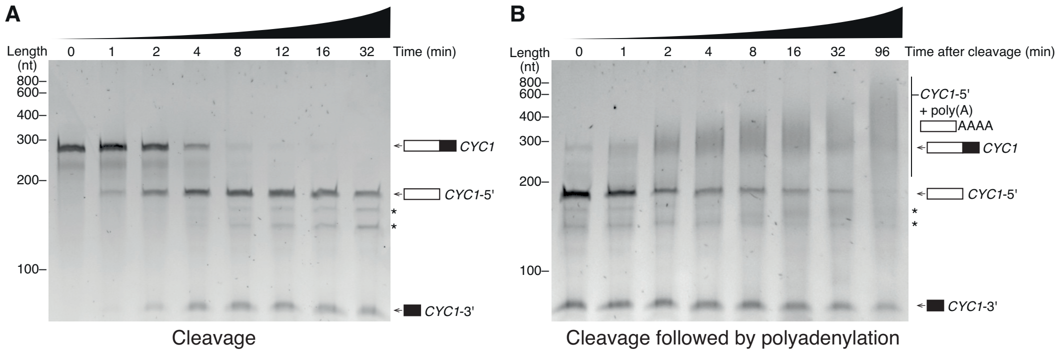

Fig. S1. Activity of native CPF. (**A**) The endonuclease activity of CPF was assayed using the *CYC1* model RNA. If ATP is left out of the reaction, polyadenylation cannot take place. (**B**) Uncoupled cleavage and polyadenylation was assayed by allowing CPF to cleave the *CYC1* RNA in the absence of ATP. After 10 minutes cleavage was complete, as shown in panel (A), and ATP was added to begin the polyadenylation assay. There is no substantial difference in the rate of cleavage or polyadenylation when the reactions are uncoupled compared to fully coupled (Fig. 1B).

A

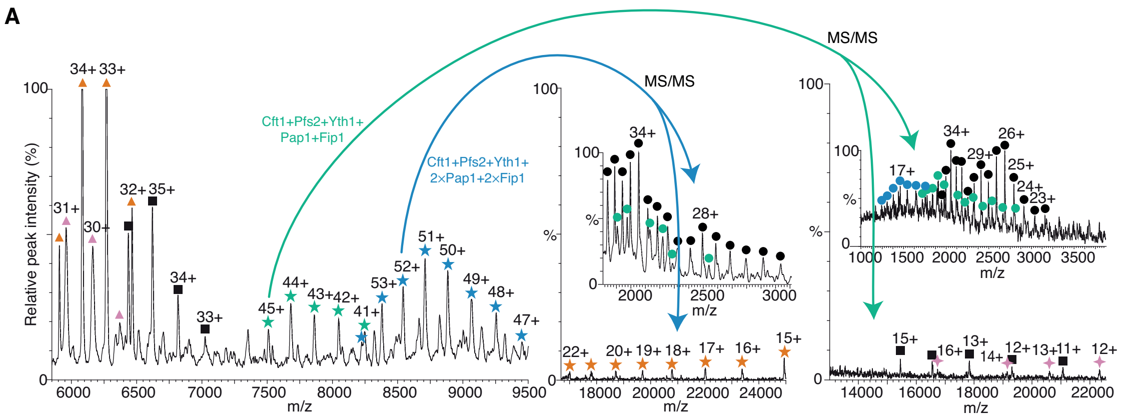

	Protein/Complex	Calculated Mass (Da)	Measured Mass (Da)
● (blue)	Yth1	24553	24807.79 ± 11.59
● (green)	Pfs2	53143	53240.72 ± 10.03
● (black)	Pap1	69843	69827.52 ± 35.93
▲ (pink)	Cft2+Ysh1	183927	184681.63 ± 42.75
▲ (orange)	Cft1+Pfs2	206548	206819.94 ± 25.15
■ (black)	Cft1+Pfs2+Yth1	231101	231647.55 ± 27.32
✦ (pink)	Cft1+Pfs2+Yth1+Fip1	266878	267987.41 ± 20.05
★ (green)	Cft1+Pfs2+Yth1+Pap1+Fip1	336720	337924.94 ± 48.14
★ (orange)	Cft1+Pfs2+Yth1+Pap1+2xFip1	372497	374023.13 ± 52.91
★ (blue)	Cft1+Pfs2+Yth1+2xPap1+2xFip1	442340	444157.66 ± 47.07

● 1 subunit
▲ 2 subunits
■ 3 subunits
✦ 4 subunits
★ 5, 6 or 7 subunits
Color is used to denote unique protein species

B

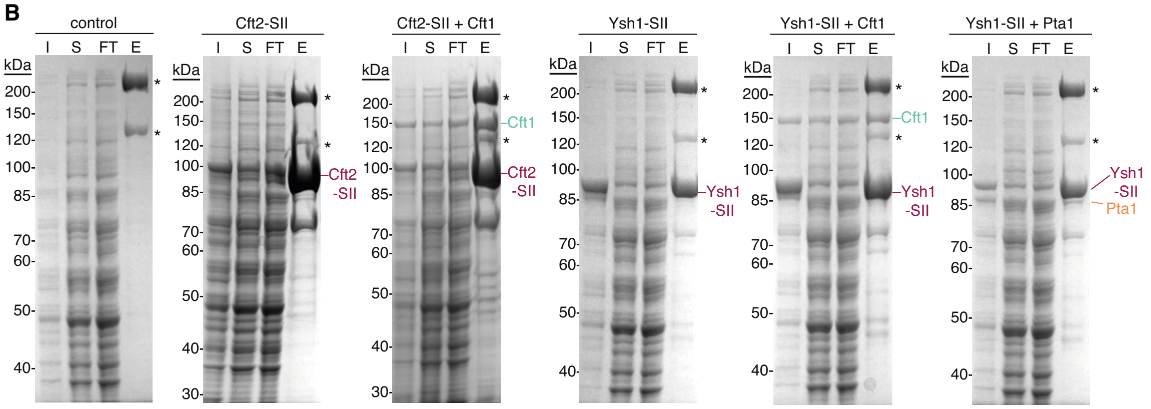

Fig. S2. Protein-protein interactions within CPF. (**A**) Representative nanoESI-MS spectra of native CPF from Pap1-TAPS yeast, showing charge states for the CPF sub-complexes (left panel). Assigned peaks are marked. Mass error (table) is the standard deviation in fitting the peaks to the charge series (see Methods). Pap1 mass includes the affinity tag. MS/MS spectra (middle and right panels) of the 45+ (7511 *m/z* parent ion, green star) and 52+ (8542 *m/z* parent ion, blue star) charge states showing dissociated subunits (1000–3500 *m/z*) and corresponding stripped complexes (14,000–24,000 *m/z*) confirm composition of the parent sub-complexes. (**B**) Pulldowns using subunits from each module (Cft1, Cft2, Ysh1 and Pta1), that were previously suggested to interact in extracts (*1*). Subunits were overexpressed in pairs with one subunit tagged, pull-downs performed, and analyzed by SDS-PAGE. This reveals potential connections between the modules (Cft1–Cft2, Cft1–Ysh1, and Ysh1–Pta1). A Ysh1–Pta1 interaction is consistent with Pta1 acting as a scaffold for CPF assembly (*5*, *70*, *71*). Control contained empty plasmids. Contaminating proteins (Acc1 and Pyc1/Pyc2) are indicated with asterisks. I is insoluble fraction; S is soluble fraction; FT is StrepTactin flow-through; E is elution.

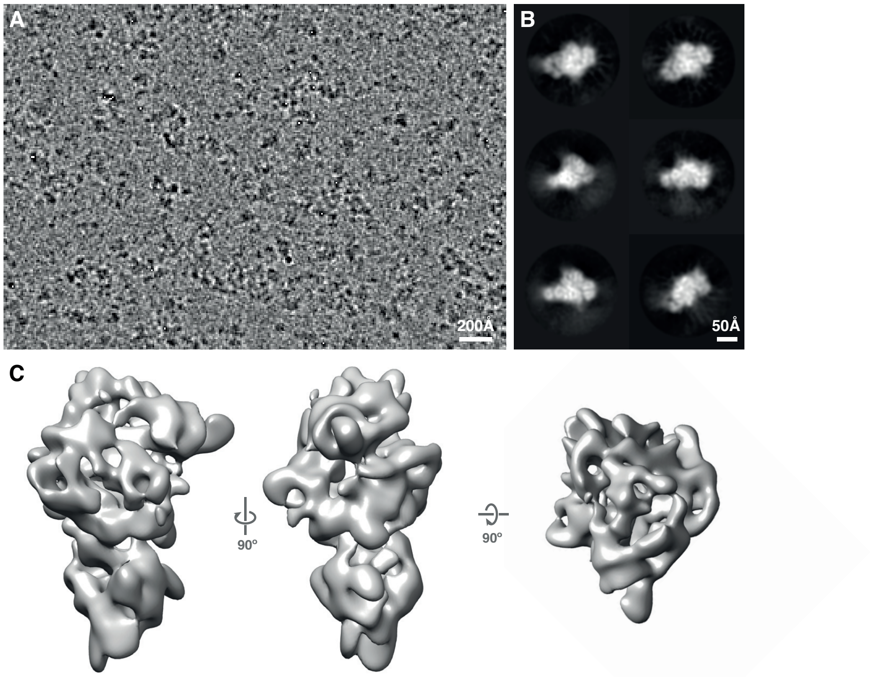

Fig. S3. Cryo-EM of crosslinked budding yeast CPF. CPF purified from Ref2-TAPS yeast was analyzed by cryo-EM. (**A**) Cryo-EM micrograph. (**B**) Reference free 2D class averages. **(C)** 3D reconstruction. This structure is ~110 Å in the longest dimension.

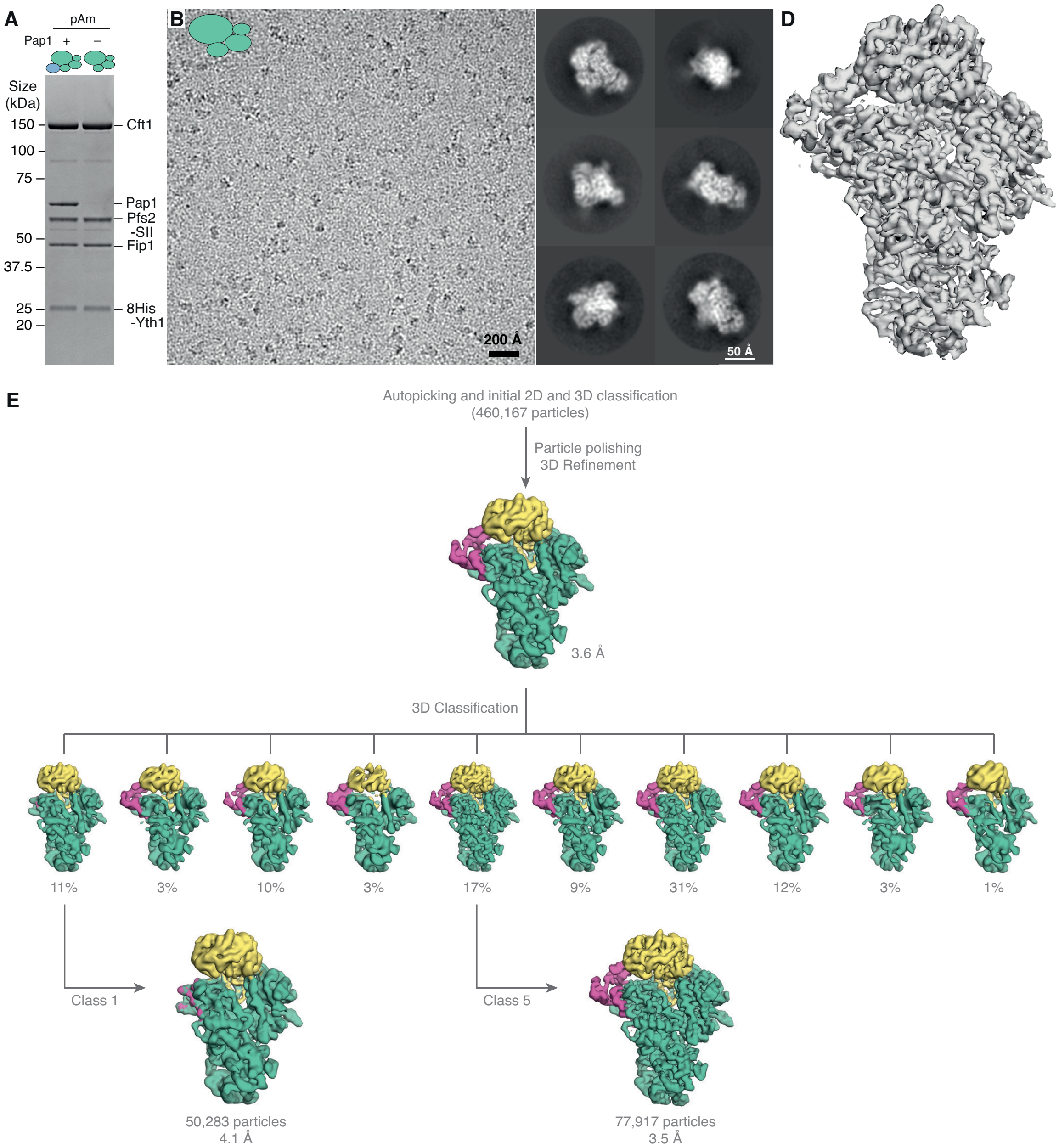

Fig. S4. Cryo-EM of recombinant polymerase module. (**A**) Coomassie-stained SDS-PAGE showing purified recombinant polymerase module (pAm) with and without Pap1. Formation of the polymerase module without Pap1 is consistent with nanoESI-MS and previous studies showing that Pap1 may not be stably bound (*3*, *18*, *27*, *72*). (**B**) Cryo-EM micrograph. (**C**) Reference free 2D class averages. (**D**) 3D reconstruction. (**E**) 3D classification scheme: Iterative 2D classification and initial 3D classification resulted in 460,167 particles that were further classified in 3D as shown here. The best class (class 5) refined to 3.5 Å resolution to yield the final map. Class 1, which was missing the density corresponding to Yth1, refined to 4.1 Å resolution. No substantial conformational changes were observed between class 1 and class 5. All nominal resolutions reported were obtained during post-processing in *RELION-2*.

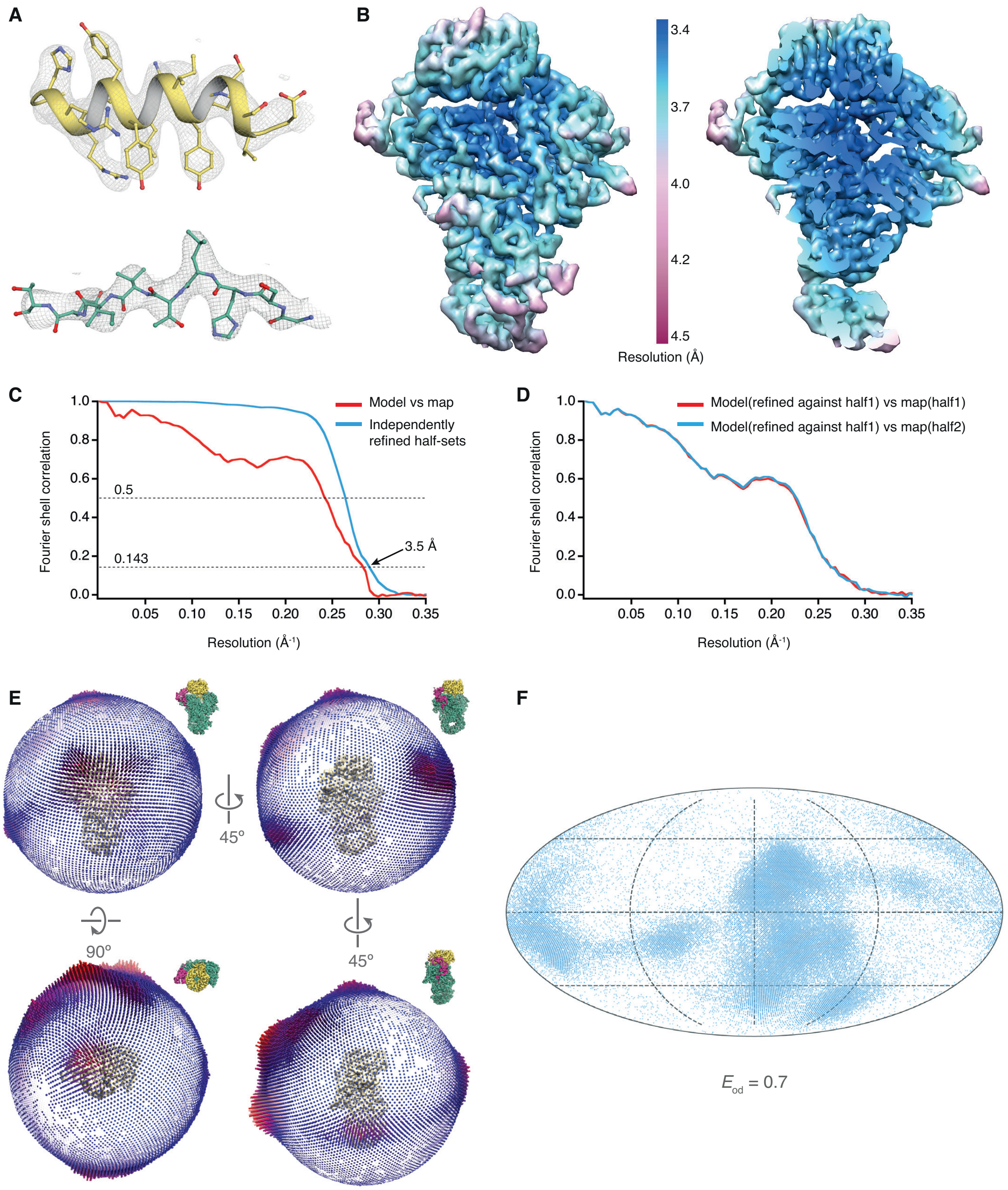

Fig. S5. Resolution assessment of polymerase module reconstruction. (**A**) Example density for alpha helix and beta strand. (**B**) Local resolution map of polymerase module. (**C-D**) Fourier shell correlation plots for gold standard refinements and model vs. map **(**C**)**, and model refined against each half map **(**D**)**. (**E-F**) Orientation distribution of 77,917 particles used in the final model, calculated using *RELION* and visualized with *Chimera* (E) or as a Mollweide projection plot (F). The 'efficiency' of particle orientation distribution, E_{od}, is 0.7 which is sufficient to reach high resolution (*73*).

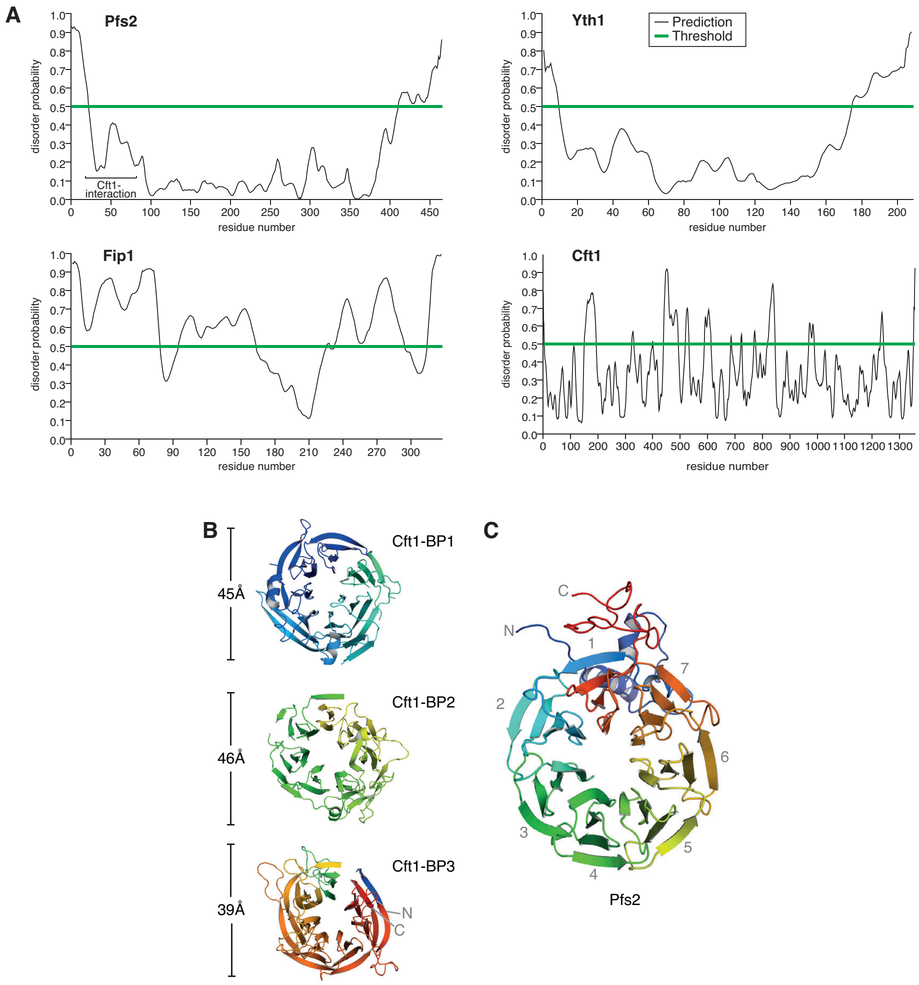

Fig. S6. Structural details of polymerase module subunits. (**A**) Plots of protein disorder prediction using *PrDOS* with a false positive rate of 5%. The N-terminal 21 amino acids of Pfs2, C-terminal ~50 amino acids of Pfs2, amino acids 151–193 and 442–493 of Cft1, and all of Fip1 are predicted to be disordered and are not observed in the cryo-EM map. The predicted disordered regions in the yeast proteins are similarly predicted to be disordered in their human counterparts. Secondary structure in the N-terminal region of Pfs2 is likely stabilized by its interaction with Cft1. The C-terminus of Pfs2 (residues 412–465) that is not visible in our structure, is disordered and is not required for viability (*3*). Similarly, the metazoan orthologue WDR33 has an extended disordered C-terminal region. Deletion of the first 25 amino acids of yeast Yth1 results in a temperature sensitive phenotype (*26*), consistent with the importance of this region for interaction with the complex. Mutation of Yth1-W70 to alanine causes Yth1 to dissociate from other CPF subunits (*25*). W70 forms pi-stacking interactions with the zinc-coordinating H85 in zinc finger 2 (Fig. 3F) and disruption of this interaction might destabilize zinc binding and association of Yth1 with CPF. All of hFIP1, like Fip1 is predicted to be disordered. (**B**) The three beta propellers from Cft1 are shown in ribbon diagram where the entire Cft1 chain is colored in rainbow from N- to C-terminus. The density for BP2 was less well defined than BP1 and BP3, and it appeared to be more flexible. (**C**) Pfs2 is shown in ribbon diagram, colored in rainbow from N- to C-terminus.

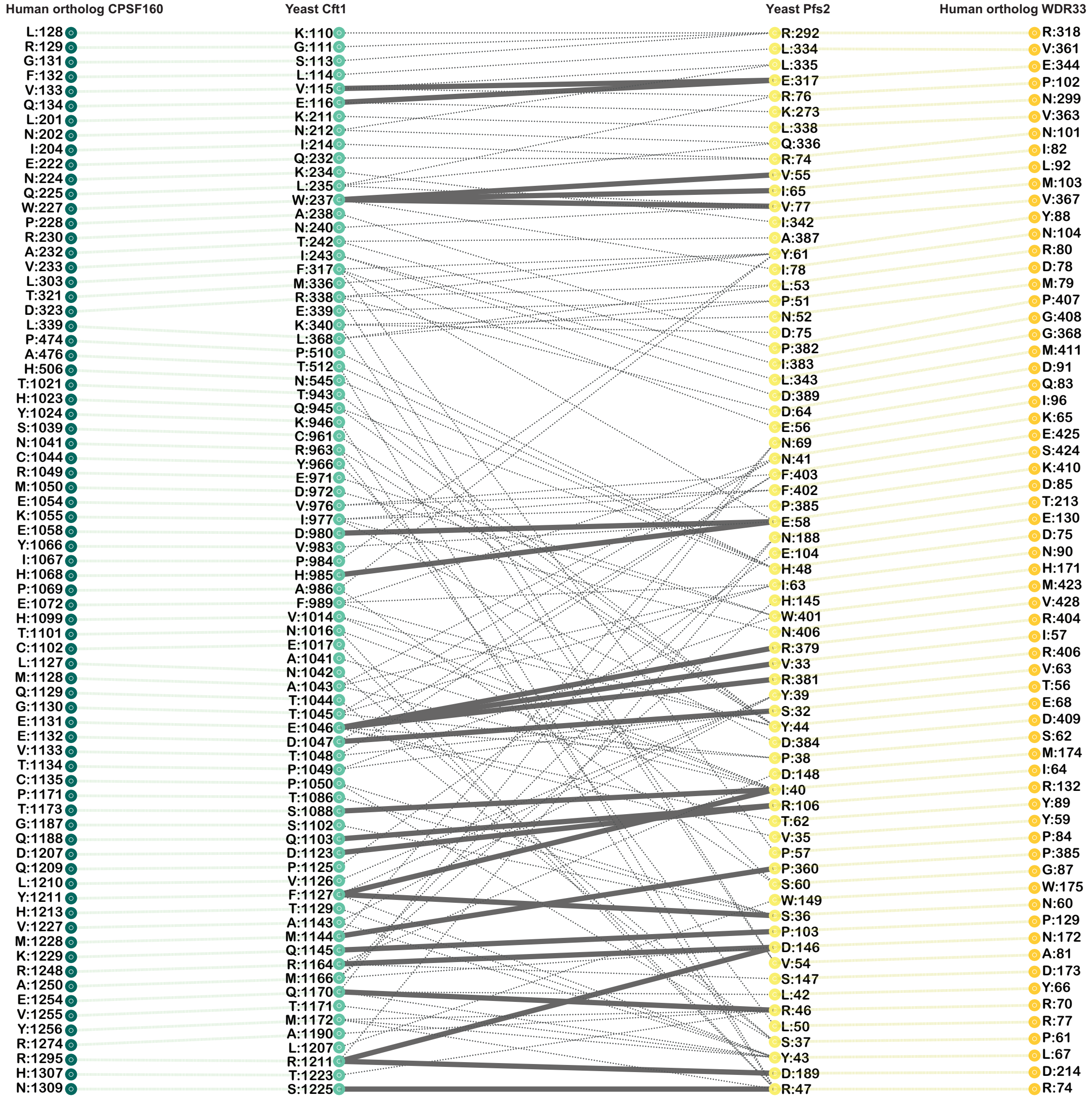

Fig. S7. Conservation of yeast Cft1–Pfs2 interactions in human CPSF160–WDR33. All residues that mediate interactions between Cft1 and Pfs2 are listed. This includes residues with salt bridges or hydrogen bonds; residues with van der Waals contacts; and hydrophobic residues whose Cα–Cα distance is greater than Cβ–Cβ (i.e. residue side chains are roughly pointing towards each other) and within 5 Å of each other (see materials and methods). The equivalent residues, indicated by orange and green dotted lines, from the human orthologs (determined from sequence alignments) are listed. Interactions that are conserved between yeast and human are shown with thick, dark grey lines. Non-conserved interactions are in thin, light grey lines. See Additional Data S1 for sequence alignments.

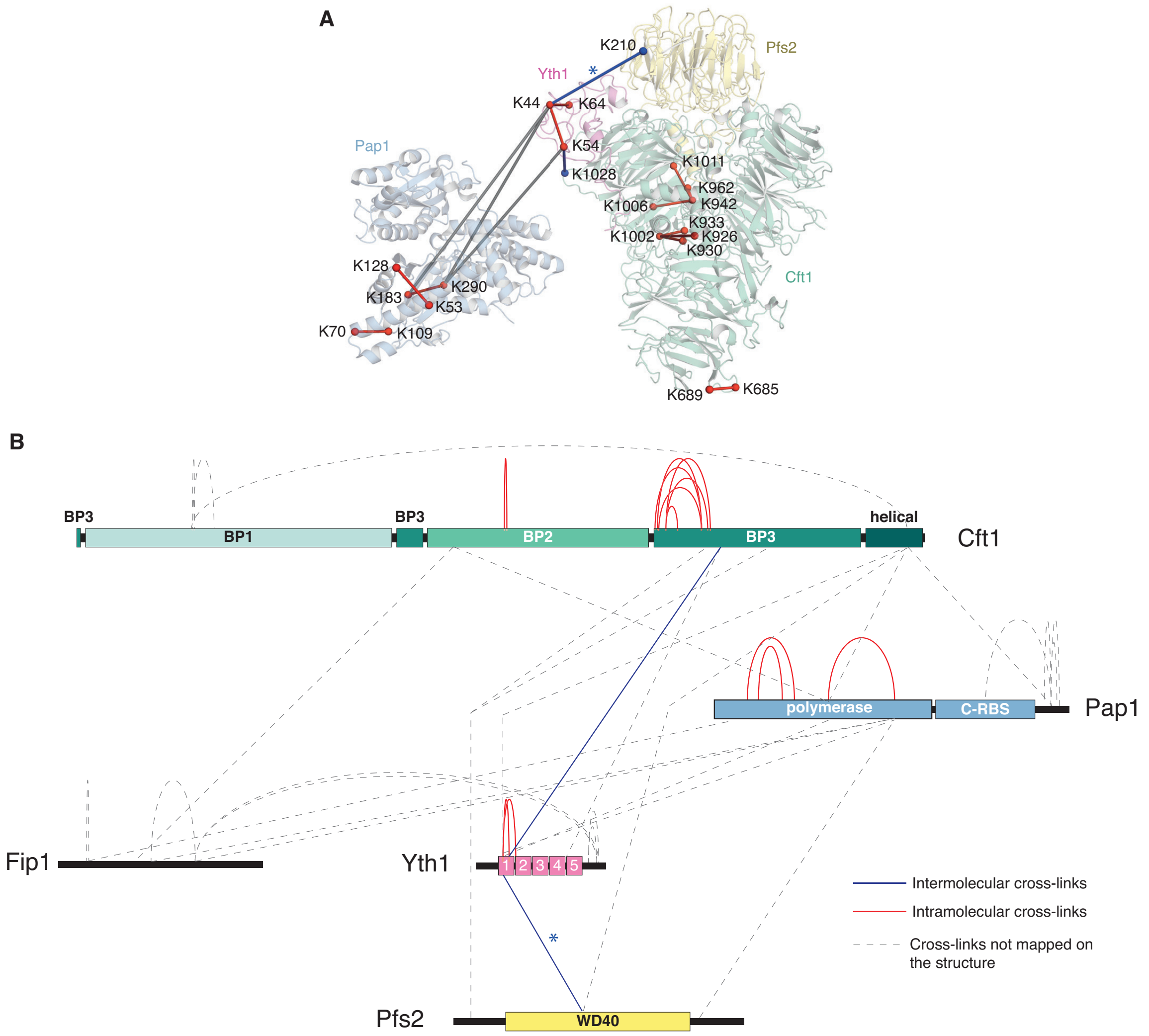

Fig. S8. Cross-linking mass spectrometry of the polymerase module. (**A**) Lysine-lysine intra- (red) and inter- (blue) protein cross-links were mapped onto the cryo-EM structure of Cft1–Pfs2–Yth1 and the crystal structure of Pap1 (PDB:2Q66). The cross-link marked with an asterisk is 33 Å, but has a similar *xQuest* score to other validated cross-links and also appears reasonable upon inspection of the structure. Lysine-lysine cross links between Pap1 and polymerase module are shown in grey. (**B**) Linkage map showing all identified lysine-lysine crosslinks. Yth1-K182, -K191 and K196 cross-link to Fip1-K219, agreeing with previous work where the C-terminal half of Yth1 was shown to bind Fip1 residues 206–220 (*14*, *25*, *26*). Fip1 (residues 80–105) also binds Pap1 (*15*). Together, these data suggest that the flexible C-terminal half of Yth1 binds the intrinsically disordered protein Fip1, which in turn interacts with Pap1 to flexibly tether it to the complex. In line with our findings, earlier work demonstrated key roles of Yth1 (zinc fingers 4 and 5) and Fip1, specifically in polyadenylation (*13*, *14*, *16*, *26*, *74*).

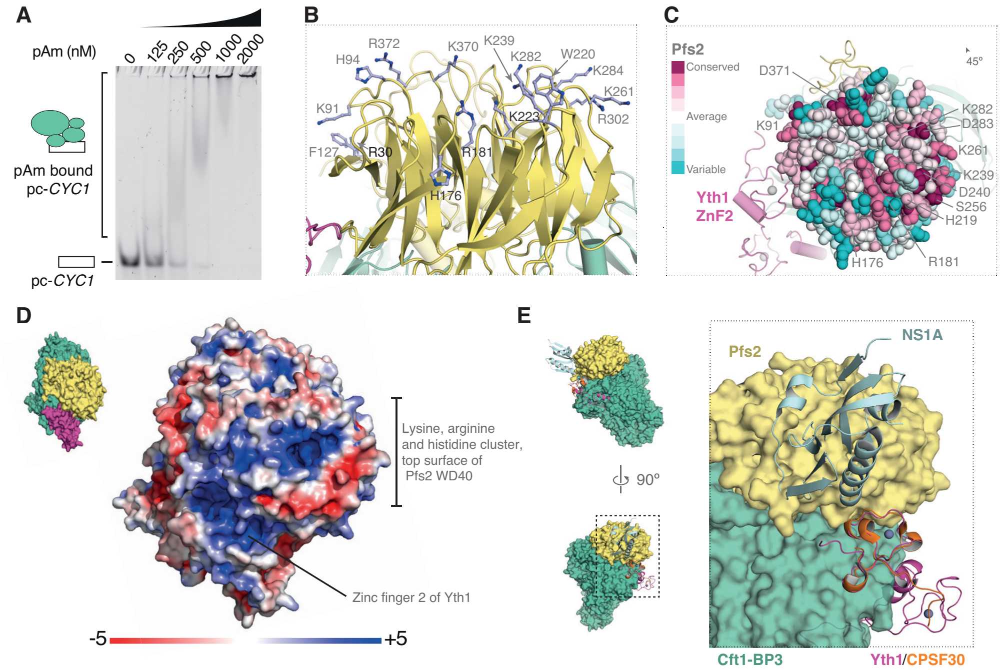

Fig. S9. The poly(A) polymerase module binds RNA. (**A**) Electrophoretic mobility shift assay (EMSA) showing that the poly(A) polymerase module binds to the 42 nt model RNA derived from the *CYC1* transcript. (**B**) In SF3b, Hsh155 and Cus1 contact RNA (*23*, *75*). UV-damaged DNA lesions are directly recognized by the DDB2 substrate receptor (*22*). The equivalent surface of Pfs2 is shown and it contains a cluster of lysine, arginine and aromatic residues (sticks). (**C**) Conservation of putative RNA binding surface across 35 Pfs2 orthologs, including human WDR33 (see Additional Data S1). (**D**) Electrostatic surface potential of the Cft1–Pfs2–Yth1 complex of the polymerase module is plotted onto the solvent-accessible surface (blue is positive charge, red is negative charge, in the range ± 5 kT/e). (**E**) The high sequence similarities of subunits and conserved Cft1–Pfs2 interactions allow us to translate our findings to the mammalian system. Specifically, in human influenza A infections, a viral protein NS1A binds to Yth1/CPSF30 to disrupt normal 3′-end processing (*10*, *19*). Our structure of the polymerase module provides mechanistic insight into how NS1A (pale cyan; PDB 2RHK) hijacks the 3′-end processing machinery: By binding to Yth1/CPSF30 zinc fingers 2 and 3 (yeast and human structures superimposed in magenta and orange, respectively), NS1A may dislodge them from Pfs2/WDR33 (yellow), and could obstruct the proposed composite RNA binding site on the polymerase module.

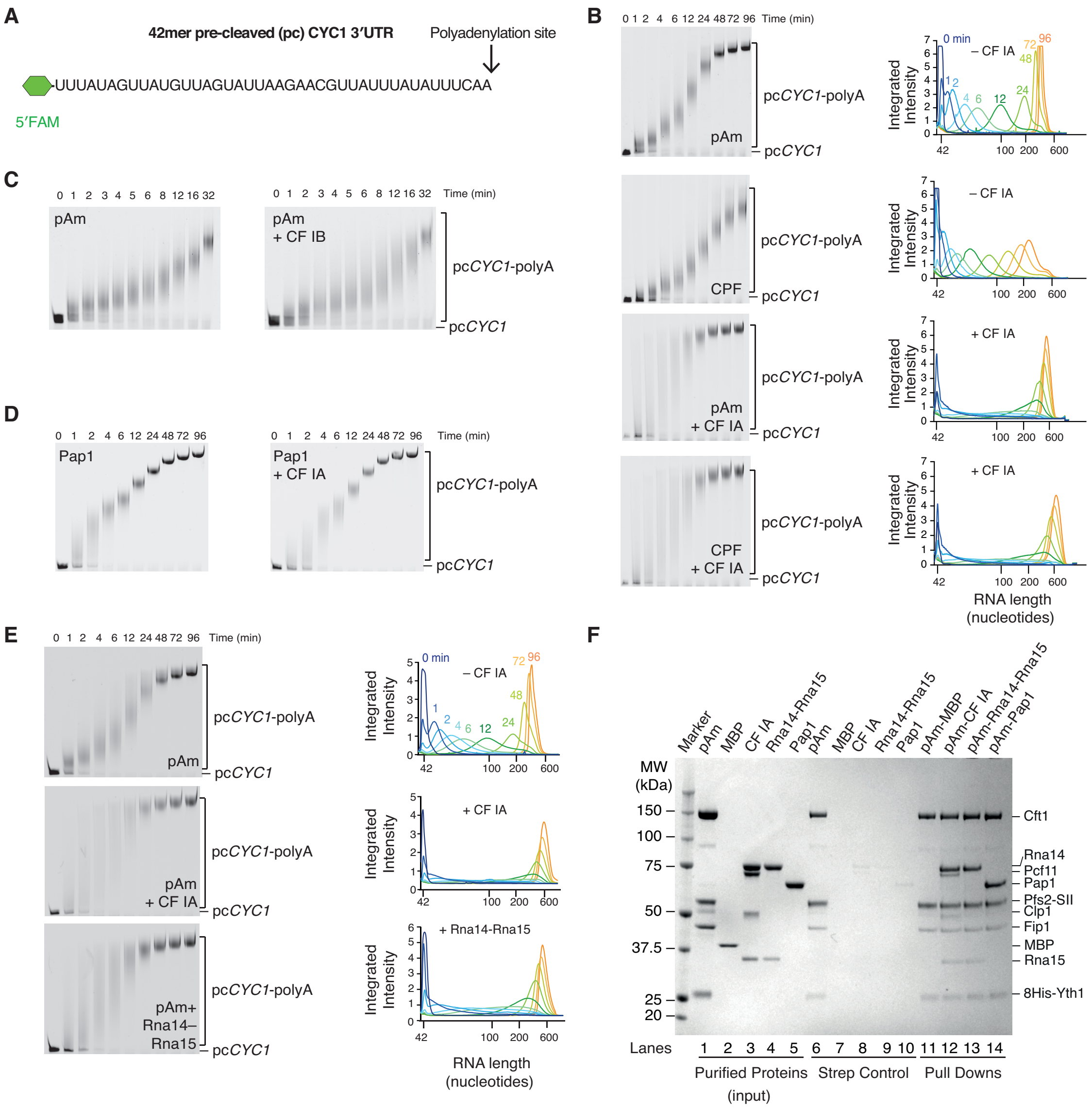

Fig. S10. Effects of CF IA and CF IB on *in vitro* polyadenylation. (**A**) Sequence of the 42 nt *CYC1* pre-cleaved (pc) RNA. (**B–E**) Polyadenylation of pc*CYC1* RNA analyzed on 15% denaturing urea PAGE. (**B**) Comparison of polymerase module (pAm) and intact CPF (with and without CF IA). Densitometric analyses of the gels are plotted on the right; RNA length is marked based on an RNA ladder. (**C**) CF IB has no substantial effect on the activity of the polymerase module. (**D**) CF IA has no substantial effect on the activity of isolated Pap1. (**E**) Rna14–Rna15 was sufficient to stimulate poly-adenylation by the polymerase module. The upper two panels in (E) are reproduced from Fig. 4D. (**F**) Coomassie-stained SDS-PAGE of pulldown experiments. Lanes 1–5: purified recombinant proteins. Lanes 6–10: magnetic streptavidin beads after incubation with the polymerase module, maltose-binding protein (MBP), CF IA, Rna14–Rna15 or Pap1. Lanes 11–14: immobilized polymerase module after incubation and pulldown with MBP, CF IA, Rna14–Rna15 or Pap1. Interaction of pAm and Rna14–Rna15 agrees with previous data suggesting Pfs2 and Fip1 bind Rna14 (*3*, *16*). CF IA may stimulate polyadenylation by contributing RNA binding sites to the complex, in agreement with a study showing that physical tethering of RNA to Pap1 increases its activity (*16*).

Table S1. Yeast CPF and human CPSF subunits.

Yeast Protein	M.W. (kDa)	Human Protein	M.W. (kDa)	% sequence identity (similarity)	Proposed role
Cft1/Yhh1	153	CPSF160/CPSF1	161	17 (34)	Scaffold
Cft2/Ydh1	96	CPSF100/CPSF2	88	22 (41)	Inactive endonuclease
Pta1	89	Symplekin	141	17 (37)	Scaffold
Ysh1/Brr5	88	CPSF73/CPSF3	77	44 (65)	Endonuclease
Pap1	65	PAPOLA	83	40 (59)	Poly(A) polymerase
Ref2	60	n.d.	-	-	Regulates Glc7
Pfs2	53	WDR33	146	37 (57)	Scaffold, RNA binding
Mpe1	50	RBBP6	202	28 (48)	RNA binding
Pti1	47	n.d.	-	-	Scaffold
Swd2/Cps35	37	WDR82	35	34 (52)	Transcription termination
Glc7	36	PP1A	38	85 (93)	Phosphatase (Pol II Y1P)
Fip1	36	hFip1	67	22 (34)	Binds Pap1
Yth1	25	CPSF30/CPSF4	30	36 (51)	RNA binding
Ssu72	24	SSU72	23	44 (65)	Phosphatase (Pol II S2P)
Syc1	21	n.d.	-	-	APT-defining subunit

n.d., none detected

Table S2. Sub-complexes of CPF from nanoESI-MS.

Assigned and verified interactions within CPF and APT used for network generation in Fig. 1C. The YJR141w/Ipa1 protein, recently shown to genetically interact with CPF (*76*), binds Ysh1 and Mpe1. However, YJR141w/Ipa1 was only found in purifications from Mpe1-tagged strains, suggesting that it is not a stable component of intact CPF. Complexes containing YJR141w or multiple copies of Pap1 or Fip1 were not included in the network analysis. All individual (isolated) subunits except Fip1 and Pta1 were also identified by nanoESI-MS.

Module	Sub-complex	Strains
Polymerase	Cft1+Pfs2	Pta1-TAPS, Mpe1-TAPS, Pap1-TAPS, Ref2-TAPS
	Cft1+Pfs2+Pap1	Pta1-TAPS, Mpe1-TAPS
	Cft1+Pfs2+Pap1+Fip1	Pap1-TAPS
	Cft1+Pfs2+Yth1	Pta1-TAPS, Mpe1-TAPS, Pap1-TAPS, Ref2-TAPS
	Cft1+Pfs2+Yth1+Fip1	Pap1-TAPS
	Cft1+Pfs2+Yth1+Pap1	Pta1-TAPS, Mpe1-TAPS, Ref2-TAPS
	Cft1+Pfs2+Yth1+Pap1+Fip1	Pta1-TAPS, Mpe1-TAPS, Pap1-TAPS
	Cft1+Yth1	Pap1-TAPS
	Cft1+Yth1+Pap1	Mpe1-TAPS
	Cft1+Yth1+Pap1+Fip1	Pap1-TAPS
	Pap1+Fip1	Pap1-TAPS
Multiple Polymerase (2 Pap1 or Fip1)	Cft1+Pfs2+Yth1+2XPap1+2XFip1	Pta1-TAPS, Pap1-TAPS
	Cft1+Pfs2+Yth1+Pap1+2XFip1	Pap1-TAPS
	Cft1+Pfs2+Yth1+2XFip1	Pap1-TAPS
	Cft1+Pfs2+2xPap1+2xFip1	Mpe1-TAPS
Nuclease	Cft2+Ysh1	Pta1-TAPS, Mpe1-TAPS, Pap1-TAPS, Ref2-TAPS
	Ysh1+Mpe1	Mpe1-TAPS
	Ysh1+YJR141w	Mpe1-TAPS
	Ysh1+Mpe1+YJR141w	Mpe1-TAPS
Phosphatase	Glc7+Ref2	Ref2-TAPS, Syc1-TAPS
	Glc7+Swd2	Ref2-TAPS, Syc1-TAPS
	Glc7+Swd2+Ref2	Ref2-TAPS
	Pta1+Ssu72	Syc1-TAPS
	Ssu72+Pti1	Syc1-TAPS
	Ssu72+Pti1+Pta1	Ref2-TAPS, Pta1-TAPS, Syc1-TAPS, Pap1-TAPS
	Ssu72+Pti1+Pta1+Glc7+Swd2+Ref2	Ref2-TAPS
	Swd2+Ref2	Ref2-TAPS
APT	Glc7+Swd2+Syc1	Syc1-TAPS
	Pta1+Syc1	Syc1-TAPS
	Pti1+Pta1+Syc1	Ref2-TAPS, Syc1-TAPS
	Pti1+Pta1+Syc1+Glc7+Swd2+Ref2	Ref2-TAPS
	Ssu72+Pta1+Syc1	Ref2-TAPS, Pta1-TAPS, Syc1-TAPS
	Ssu72+Pti1+Pta1+Syc1	Ref2-TAPS, Pta1-TAPS, Syc1-TAPS
	Ssu72+Pti1+Pta1+Syc1+Glc7+Swd2	Ref2-TAPS
	Ssu72+Pti1+Pta1+Syc1+Glc7+Swd2+Ref2	Syc1-TAPS
	Ssu72+Pti1+Pta1+Syc1+Swd2+Ref2	Ref2-TAPS
	Syc1+Glc7	Syc1-TAPS
	Syc1+Glc7+Swd2+Ref2	Syc1-TAPS

Table S3. Data collection and refinement statistics for polymerase module cryo-EM.

**Data collection**	
Pixel size (Å)	1.4
Defocus range (μm)	1.5 – 3.5
Voltage (keV)	300
Electron dose (e-/ Å^2)	45
No. of particles	77,917
Efficiency (E_{od})	0.7
**Model composition**	
Non-hydrogen atoms	13,688
Protein residues	1,715
Zinc ions	2
**Model refinement**	
Resolution (Å)	3.5
Average B-factor (Å^2)	-120
Fourier shell correlation	0.80
Rfactor	0.39
**RMS deviations**	
Bonds (Å)	0.0066
Angles (°)	1.22
**Validation**	
Molprobity score	2.62*
Clashscore, all atoms	9.50**
Good rotamers (%)	95.14
**Ramachandran plot**	
Favoured (%)	88.16
Outliers (%)	1.6

(*) 97st percentile (N=342, 3.50Å ± 0.25Å)
(**) 97th percentile (N=37, 3Å - 9999Å)

Table S4. List of amino acids involved in protein–protein cross-linking, identified by mass spectrometry.

Protein 1	Protein 2	Distance (Å)	pAm	pAm + Pap1	
Cft1_K685	Cft1_K689	9.8	✓	×	Intra-protein crosslinks mapped on to the structure
Cft1_K842	Cft1_Y1266	22.3	✓	✓	
Cft1_K926	Cft1_K1002	27.4	✓	×	
Cft1_K930	Cft1_K1002	15.0	✓	✓	
Cft1_K930	Cft1_S1000	13.7	✓	×	
Cft1_K933	Cft1_K1002	9.3	✓	×	
Cft1_Y941	Cft1_K1006	9.8	✓	×	
Cft1_K942	Cft1_S959	10.1	×	✓	
Cft1_K942	Cft1_K962	5.2	✓	×	
Cft1_K942	Cft1_K1006	13.2	×	✓	
Cft1_K942	Cft1_K1011	14.7	✓	✓	
Cft1_S959	Cft1_K1006	9.3	✓	×	
Cft1_K942	Cft1_S1013	14.6	✓	×	
Yth1_K44	Yth1_K54	15	✓	✓	
Yth1_K44	Yth1_K64	23.5	✓	×	
Pap1_K53	Pap1_K128	15	×	✓	
Pap1_K70	Pap1_K109	11.8	×	✓	
Pap1_K183	Pap1_K290	27	×	✓	
Cft1_S1027	Yth1_K54	16	✓	✓	Inter-protein crosslinks mapped on to the structure
Cft1_K1028	Yth1_K54	15.3	✓	✓	
Pfs2_K210	Yth1_K44	32.8	✓	×	
Cft1_K184	Cft1_K189	N/A	✓	×	
Cft1_K184	Cft1_K224	N/A	×	✓	
Cft1_K184	Cft1_K1330	N/A	✓	✓	
Cft1_K186	Cft1_S198	N/A	✓	✓	
Cft1_K189	Cft1_S198	N/A	×	✓	
Cft1_S416	Cft1_K440	N/A	✓	×	
Cft1_S591	Cft1_K609	N/A	✓	✓	
Cft1_Y585	Pap1_K5	N/A	×	✓	
Cft1_K609	Pap1_K183	N/A	×	✓	
Cft1_K1330	Pap1_K183	N/A	×	✓	
Cft1_K1325	Pap1_K536	N/A	×	✓	
Cft1_K1011	Pfs2_K25	N/A	✓	×	
Cft1_K1104	Pfs2_K25	N/A	✓	×	
Cft1_K1104	Pfs2_Y26	N/A	✓	×	
Cft1_K1330	Pfs2_S207	N/A	✓	×	
Cft1_K1330	Pfs2_K210	N/A	×	✓	
Cft1_K1330	Pfs2_Y393	N/A	✓	×	
Cft1_S1027	Yth1_K145	N/A	✓	×	
Cft1_K1028	Yth1_K145	N/A	✓	×	
Cft1_K1330	Yth1_K44	N/A	✓	✓	
Cft1_S591	Fip1_K44	N/A	✓	×	
Cft1_S591	Fip1_K47	N/A	✓	×	
Cft1_S591	Fip1_K148	N/A	✓	✓	
Cft1_K609	Fip1_K126	N/A	×	✓	
Cft1_S1027	Fip1_K148	N/A	×	✓	
Cft1_S1349	Fip1_K148	N/A	✓	×	
Pap1_K34	Pap1_S38	N/A	×	✓	
Pap1_S344	Pap1_K536	N/A	×	✓	
Pap1_K527	Pap1_K536	N/A	×	✓	
Pap1_K432	Pap1_K536	N/A	×	✓	
Pap1_K536	Pap1_K546	N/A	×	✓	
Pap1_K536	Pap1_K549	N/A	×	✓	
Pap1_K536	Pap1_S550	N/A	×	✓	
Pap1_K290	Pfs2_Y393	N/A	×	✓	
Pap1_K290	Pfs2_K396	N/A	×	✓	
Pap1_K183	Yth1_K44	N/A	×	✓	
Pap1_K290	Yth1_K44	N/A	×	✓	
Pap1_K290	Yth1_54	N/A	×	✓	
Pap1_K34	Fip1_K44	N/A	×	✓	
Pap1_K290	Fip1_S121	N/A	×	✓	
Pap1_K290	Fip1_K148	N/A	×	✓	
Pap1_K290	Fip1_K219	N/A	×	✓	
Pap1_K292	Fip1_K148	N/A	×	✓	
Pfs2_Y26	Yth1_K76	N/A	✓	✓	
Pfs2_K244	Yth1_K44	54.4	✓	×	
Pfs2_S453	Yth1_K76	N/A	✓	✓	
Fip1_S38	Fip1_K47	N/A	✓	✓	
Fip1_K44	Fip1_K47	N/A	✓	✓	
Fip1_K44	Fip1_S50	N/A	✓	×	
Fip1_K148	Fip1_K219	N/A	×	✓	
Fip1_S152	Fip1_K219	N/A	✓	×	
Fip1_K219	Yth1_K182	N/A	✓	✓	
Fip1_K219	Yth1_K191	N/A	✓	✓	
Fip1_K219	Yth1_K196	N/A	✓	✓	
Yth1_K182	Yth1_K191	N/A	✓	×	
Yth1_K182	Yth1_K196	N/A	✓	✓	

Movie S1. Overall structure of the polymerase module, showing the tight interactions between the Cft1 (green), Pfs2 (yellow) and Yth1 (magenta) subunits of CPF. Cft1 is composed of three intertwined beta-propellers (BP1, BP2 and BP3) and an alpha helical C-terminal domain. The CCCH coordination of zinc fingers (ZnF) 1 and 2 of Yth1 is shown. Yth1 folds around and into the centre of Cft1-BP3 and is stabilized by electrostatic and hydrophobic interactions with both Cft1 and Pfs2. Pfs2 is stably bound to Cft1 by insertion of the Pfs2 N-terminal helices into the central groove of Cft1.

Additional Data S1

Sequence alignment of Pfs2 across major eukaryotic lineages with at least one representative from each lineage, constructed using *MSAProbs* and refined using *BLAST* and *HMMER* sequence profiles.

References and Notes

1. J. Zhao, M. M. Kessler, C. L. Moore, Cleavage factor II of *Saccharomyces cerevisiae* contains homologues to subunits of the mammalian Cleavage/ polyadenylation specificity factor and exhibits sequence-specific, ATP-dependent interaction with precursor RNA. *J. Biol. Chem.* **272**, 10831–10838 (1997). doi:10.1074/jbc.272.16.10831 Medline

2. P. J. Preker, M. Ohnacker, L. Minvielle-Sebastia, W. Keller, A multisubunit 3′ end processing factor from yeast containing poly(A) polymerase and homologues of the subunits of mammalian cleavage and polyadenylation specificity factor. *EMBO J.* **16**, 4727–4737 (1997). doi:10.1093/emboj/16.15.4727 Medline

3. M. Ohnacker, S. M. Barabino, P. J. Preker, W. Keller, The WD-repeat protein pfs2p bridges two essential factors within the yeast pre-mRNA 3′-end-processing complex. *EMBO J.* **19**, 37–47 (2000). doi:10.1093/emboj/19.1.37 Medline

4. A.-C. Gavin, M. Bösche, R. Krause, P. Grandi, M. Marzioch, A. Bauer, J. Schultz, J. M. Rick, A.-M. Michon, C.-M. Cruciat, M. Remor, C. Höfert, M. Schelder, M. Brajenovic, H. Ruffner, A. Merino, K. Klein, M. Hudak, D. Dickson, T. Rudi, V. Gnau, A. Bauch, S. Bastuck, B. Huhse, C. Leutwein, M.-A. Heurtier, R. R. Copley, A. Edelmann, E. Querfurth, V. Rybin, G. Drewes, M. Raida, T. Bouwmeester, P. Bork, B. Seraphin, B. Kuster, G. Neubauer, G. Superti-Furga, Functional organization of the yeast proteome by systematic analysis of protein complexes. *Nature* **415**, 141–147 (2002). doi:10.1038/415141a Medline

5. E. Nedea, X. He, M. Kim, J. Pootoolal, G. Zhong, V. Canadien, T. Hughes, S. Buratowski, C. L. Moore, J. Greenblatt, Organization and function of APT, a subcomplex of the yeast cleavage and polyadenylation factor involved in the formation of mRNA and small nucleolar RNA 3′-ends. *J. Biol. Chem.* **278**, 33000–33010 (2003). doi:10.1074/jbc.M304454200 Medline

6. X. He, C. Moore, Regulation of yeast mRNA 3′ end processing by phosphorylation. *Mol. Cell* **19**, 619–629 (2005). doi:10.1016/j.molcel.2005.07.016 Medline

7. A. Schreieck, A. D. Easter, S. Etzold, K. Wiederhold, M. Lidschreiber, P. Cramer, L. A. Passmore, RNA polymerase II termination involves C-terminal-domain tyrosine dephosphorylation by CPF subunit Glc7. *Nat. Struct. Mol. Biol.* **21**, 175–179 (2014). doi:10.1038/nsmb.2753 Medline

8. S. Danckwardt, M. W. Hentze, A. E. Kulozik, 3′ end mRNA processing: Molecular mechanisms and implications for health and disease. *EMBO J.* **27**, 482–498 (2008). doi:10.1038/sj.emboj.7601932 Medline

9. C. Mayr, D. P. Bartel, Widespread shortening of 3'UTRs by alternative cleavage and polyadenylation activates oncogenes in cancer cells. *Cell* **138**, 673–684 (2009). doi:10.1016/j.cell.2009.06.016 Medline

10. M. E. Nemeroff, S. M. Barabino, Y. Li, W. Keller, R. M. Krug, Influenza virus NS1 protein interacts with the cellular 30 kDa subunit of CPSF and inhibits 3'end formation of cellular pre-mRNAs. *Mol. Cell* **1**, 991–1000 (1998). doi:10.1016/S1097-2765(00)80099-4 Medline

11. H. Hernández, C. V. Robinson, Determining the stoichiometry and interactions of macromolecular assemblies from mass spectrometry. *Nat. Protoc.* **2**, 715–726 (2007). doi:10.1038/nprot.2007.73 Medline

12. A. Zhelkovsky, Y. Tacahashi, T. Nasser, X. He, U. Sterzer, T. H. Jensen, H. Domdey, C. Moore, The role of the Brr5/Ysh1 C-terminal domain and its homolog Syc1 in mRNA 3′-end processing in *Saccharomyces cerevisiae*. *RNA* **12**, 435–445 (2006). doi:10.1261/rna.2267606 Medline

13. P. J. Preker, J. Lingner, L. Minvielle-Sebastia, W. Keller, The FIP1 gene encodes a component of a yeast pre-mRNA polyadenylation factor that directly interacts with poly(A) polymerase. *Cell* **81**, 379–389 (1995). doi:10.1016/0092-8674(95)90391-7 Medline

14. S. Helmling, A. Zhelkovsky, C. L. Moore, Fip1 regulates the activity of Poly(A) polymerase through multiple interactions. *Mol. Cell. Biol.* **21**, 2026–2037 (2001). doi:10.1128/MCB.21.6.2026-2037.2001 Medline

15. G. Meinke, C. Ezeokonkwo, P. Balbo, W. Stafford, C. Moore, A. Bohm, Structure of yeast poly(A) polymerase in complex with a peptide from Fip1, an intrinsically disordered protein. *Biochemistry* **47**, 6859–6869 (2008). doi:10.1021/bi800204k Medline

16. C. Ezeokonkwo, A. Zhelkovsky, R. Lee, A. Bohm, C. L. Moore, A flexible linker region in Fip1 is needed for efficient mRNA polyadenylation. *RNA* **17**, 652–664 (2011). doi:10.1261/rna.2273111 Medline

17. K. G. Murthy, J. L. Manley, The 160-kD subunit of human cleavage-polyadenylation specificity factor coordinates pre-mRNA 3′-end formation. *Genes Dev.* **9**, 2672–2683 (1995). doi:10.1101/gad.9.21.2672 Medline

18. L. Schönemann, U. Kühn, G. Martin, P. Schäfer, A. R. Gruber, W. Keller, M. Zavolan, E. Wahle, Reconstitution of CPSF active in polyadenylation: Recognition of the polyadenylation signal by WDR33. *Genes Dev.* **28**, 2381–2393 (2014). doi:10.1101/gad.250985.114 Medline

19. K. Das, L. C. Ma, R. Xiao, B. Radvansky, J. Aramini, L. Zhao, J. Marklund, R. L. Kuo, K. Y. Twu, E. Arnold, R. M. Krug, G. T. Montelione, Structural basis for suppression of a host antiviral response by influenza A virus. *Proc. Natl. Acad. Sci. U.S.A.* **105**, 13093–13098 (2008). Medline

20. J. Bard, A. M. Zhelkovsky, S. Helmling, T. N. Earnest, C. L. Moore, A. Bohm, Structure of yeast poly(A) polymerase alone and in complex with 3′-dATP. *Science* **289**, 1346–1349 (2000). doi:10.1126/science.289.5483.1346 Medline

21. T. Li, X. Chen, K. C. Garbutt, P. Zhou, N. Zheng, Structure of DDB1 in complex with a paramyxovirus V protein: Viral hijack of a propeller cluster in ubiquitin ligase. *Cell* **124**, 105–117 (2006). doi:10.1016/j.cell.2005.10.033 Medline

22. A. Scrima, R. Konícková, B. K. Czyzewski, Y. Kawasaki, P. D. Jeffrey, R. Groisman, Y. Nakatani, S. Iwai, N. P. Pavletich, N. H. Thomä, Structural basis of UV DNA-damage recognition by the DDB1-DDB2 complex. *Cell* **135**, 1213–1223 (2008). doi:10.1016/j.cell.2008.10.045 Medline

23. C. Yan, R. Wan, R. Bai, G. Huang, Y. Shi, Structure of a yeast activated spliceosome at 3.5 Å resolution. *Science* **353**, 904–911 (2016). doi:10.1126/science.aag0291 Medline

24. C. Cretu, J. Schmitzová, A. Ponce-Salvatierra, O. Dybkov, E. I. De Laurentiis, K. Sharma, C. L. Will, H. Urlaub, R. Lührmann, V. Pena, Molecular Architecture of SF3b and structural consequences of its cancer-related mutations. *Mol. Cell* **64**, 307–319 (2016). doi:10.1016/j.molcel.2016.08.036 Medline

25. S. M. Barabino, M. Ohnacker, W. Keller, Distinct roles of two Yth1p domains in 3′-end cleavage and polyadenylation of yeast pre-mRNAs. *EMBO J.* **19**, 3778–3787 (2000). doi:10.1093/emboj/19.14.3778 Medline

26. Y. Tacahashi, S. Helmling, C. L. Moore, Functional dissection of the zinc finger and flanking domains of the Yth1 cleavage/polyadenylation factor. *Nucleic Acids Res.* **31**, 1744–1752 (2003). doi:10.1093/nar/gkg265 Medline

27. S. L. Chan, I. Huppertz, C. Yao, L. Weng, J. J. Moresco, J. R. Yates 3rd, J. Ule, J. L. Manley, Y. Shi, CPSF30 and Wdr33 directly bind to AAUAAA in mammalian mRNA 3′ processing. *Genes Dev.* **28**, 2370–2380 (2014). doi:10.1101/gad.250993.114 Medline

28. L. Minvielle-Sebastia, P. J. Preker, W. Keller, RNA14 and RNA15 proteins as components of a yeast pre-mRNA 3′-end processing factor. *Science* **266**, 1702–1705 (1994). doi:10.1126/science.7992054 Medline

29. M. M. Kessler, J. Zhao, C. L. Moore, Purification of the *Saccharomyces cerevisiae* cleavage/polyadenylation factor I. Separation into two components that are required for both cleavage and polyadenylation of mRNA 3′ ends. *J. Biol. Chem.* **271**, 27167–27175 (1996). doi:10.1074/jbc.271.43.27167 Medline

30. M. M. Kessler, M. F. Henry, E. Shen, J. Zhao, S. Gross, P. A. Silver, C. L. Moore, Hrp1, a sequence-specific RNA-binding protein that shuttles between the nucleus and the cytoplasm, is required for mRNA 3′-end formation in yeast. *Genes Dev.* **11**, 2545–2556 (1997). doi:10.1101/gad.11.19.2545 Medline

31. J. M. B. Gordon, S. Shikov, J. N. Kuehner, M. Liriano, E. Lee, W. Stafford, M. B. Poulsen, C. Harrison, C. Moore, A. Bohm, Reconstitution of CF IA from overexpressed subunits reveals stoichiometry and provides insights into molecular topology. *Biochemistry* **50**, 10203–10214 (2011). doi:10.1021/bi200964p Medline

32. I. V. Chernushevich, B. A. Thomson, Collisional cooling of large ions in electrospray mass spectrometry. *Anal. Chem.* **76**, 1754–1760 (2004). doi:10.1021/ac035406j Medline

33. F. Sobott, H. Hernández, M. G. McCammon, M. A. Tito, C. V. Robinson, A tandem mass spectrometer for improved transmission and analysis of large macromolecular assemblies. *Anal. Chem.* **74**, 1402–1407 (2002). doi:10.1021/ac0110552 Medline

34. F. Sobott, M. G. McCammon, H. Hernández, C. V. Robinson, The flight of macromolecular complexes in a mass spectrometer. *Philos. Trans. A Math. Phys. Eng. Sci.* **363**, 379–389, discussion 389–391 (2005). Medline

35. A. Laganowsky, E. Reading, J. T. S. Hopper, C. V. Robinson, Mass spectrometry of intact membrane protein complexes. *Nat. Protoc.* **8**, 639–651 (2013). doi:10.1038/nprot.2013.024 Medline

36. N. Zhang, Y. Gordiyenko, N. Joly, E. Lawton, C. V. Robinson, M. Buck, Subunit dynamics and nucleotide-dependent asymmetry of an AAA(+) transcription complex. *J. Mol. Biol.* **426**, 71–83 (2014). doi:10.1016/j.jmb.2013.08.018 Medline

37. N. Morgner, C. V. Robinson, Massign: An assignment strategy for maximizing information from the mass spectra of heterogeneous protein assemblies. *Anal. Chem.* **84**, 2939–2948 (2012). doi:10.1021/ac300056a Medline

38. T. Taverner, H. Hernández, M. Sharon, B. T. Ruotolo, D. Matak-Vinković, D. Devos, R. B. Russell, C. V. Robinson, Subunit architecture of intact protein complexes from mass spectrometry and homology modeling. *Acc. Chem. Res.* **41**, 617–627 (2008). doi:10.1021/ar700218q Medline

39. W. P. Galej, C. Oubridge, A. J. Newman, K. Nagai, Crystal structure of Prp8 reveals active site cavity of the spliceosome. *Nature* **493**, 638–643 (2013). doi:10.1038/nature11843 Medline

40. C. Bieniossek, T. J. Richmond, I. Berger, MultiBac: multigene baculovirus-based eukaryotic protein complex production. *Curr Protoc Protein Sci*. **Chapter 5**, Unit 5.20 (2008).

41. J. A. W. Stowell, M. W. Webster, A. Kögel, J. Wolf, K. L. Shelley, L. A. Passmore, Reconstitution of targeted deadenylation by the Ccr4-Not complex and the YTH domain protein Mmi1. *Cell Reports* **17**, 1978–1989 (2016). doi:10.1016/j.celrep.2016.10.066 Medline

42. C. J. Russo, L. A. Passmore, Electron microscopy: Ultrastable gold substrates for electron cryomicroscopy. *Science* **346**, 1377–1380 (2014). doi:10.1126/science.1259530 Medline

43. R. S. Pantelic, J. C. Meyer, U. Kaiser, W. Baumeister, J. M. Plitzko, Graphene oxide: A substrate for optimizing preparations of frozen-hydrated samples. *J. Struct. Biol.* **170**, 152–156 (2010). doi:10.1016/j.jsb.2009.12.020 Medline

44. T. G. Martin, A. Boland, A. W P Fitzpatrick, S. H. W. Scheres, Graphene Oxide Grid Preparation. figshare. https://dx.doi.org/10.6084/m9.figshare.3178669.v1 (2016).

45. X. Li, P. Mooney, S. Zheng, C. R. Booth, M. B. Braunfeld, S. Gubbens, D. A. Agard, Y. Cheng, Electron counting and beam-induced motion correction enable near-atomic-resolution single-particle cryo-EM. *Nat. Methods* **10**, 584–590 (2013). doi:10.1038/nmeth.2472 Medline

46. K. Zhang, Gctf: Real-time CTF determination and correction. *J. Struct. Biol.* **193**, 1–12 (2016). doi:10.1016/j.jsb.2015.11.003 Medline

47. S. H. W. Scheres, RELION: Implementation of a Bayesian approach to cryo-EM structure determination. *J. Struct. Biol.* **180**, 519–530 (2012). doi:10.1016/j.jsb.2012.09.006 Medline

48. D. Kimanius, B. O. Forsberg, S. H. Scheres, E. Lindahl, Accelerated cryo-EM structure determination with parallelisation using GPUs in RELION-2. *eLife* **5**, 19 (2016). doi:10.7554/eLife.18722 Medline

49. H. Elmlund, D. Elmlund, S. Bengio, PRIME: Probabilistic initial 3D model generation for single-particle cryo-electron microscopy. *Structure* **21**, 1299–1306 (2013). doi:10.1016/j.str.2013.07.002 Medline

50. S. Q. Zheng, E. Palovcak, J.-P. Armache, K. A. Verba, Y. Cheng, D. A. Agard, MotionCor2: Anisotropic correction of beam-induced motion for improved cryo-electron microscopy. *Nat. Methods* **14**, 331–332 (2017). doi:10.1038/nmeth.4193 Medline

51. L. A. Kelley, S. Mezulis, C. M. Yates, M. N. Wass, M. J. E. Sternberg, The Phyre2 web portal for protein modeling, prediction and analysis. *Nat. Protoc.* **10**, 845–858 (2015). doi:10.1038/nprot.2015.053 Medline

52. P. Emsley, B. Lohkamp, W. G. Scott, K. Cowtan, Features and development of Coot. *Acta Crystallogr. D Biol. Crystallogr.* **66**, 486–501 (2010). doi:10.1107/S0907444910007493 Medline

53. A. Brown, F. Long, R. A. Nicholls, J. Toots, P. Emsley, G. Murshudov, Tools for macromolecular model building and refinement into electron cryo-microscopy reconstructions. *Acta Crystallogr. D Biol. Crystallogr.* **71**, 136–153 (2015). doi:10.1107/S1399004714021683 Medline

54. R. A. Nicholls, F. Long, G. N. Murshudov, Low-resolution refinement tools in REFMAC5. *Acta Crystallogr. D Biol. Crystallogr.* **68**, 404–417 (2012). doi:10.1107/S090744491105606X Medline

55. P. V. Afonine, R. W. Grosse-Kunstleve, N. Echols, J. J. Headd, N. W. Moriarty, M. Mustyakimov, T. C. Terwilliger, A. Urzhumtsev, P. H. Zwart, P. D. Adams, Towards automated crystallographic structure refinement with phenix.refine. *Acta Crystallogr. D Biol. Crystallogr.* **68**, 352–367 (2012). doi:10.1107/S0907444912001308 Medline

56. V. B. Chen, W. B. Arendall 3rd, J. J. Headd, D. A. Keedy, R. M. Immormino, G. J. Kapral, L. W. Murray, J. S. Richardson, D. C. Richardson, MolProbity: All-atom structure validation for macromolecular crystallography. *Acta Crystallogr. D Biol. Crystallogr.* **66**, 12–21 (2010). doi:10.1107/S0907444909042073 Medline

57. E. F. Pettersen, T. D. Goddard, C. C. Huang, G. S. Couch, D. M. Greenblatt, E. C. Meng, T. E. Ferrin, UCSF Chimera—a visualization system for exploratory research and analysis. *J. Comput. Chem.* **25**, 1605–1612 (2004). doi:10.1002/jcc.20084 Medline

58. M. Schmid, M. B. Poulsen, P. Olszewski, V. Pelechano, C. Saguez, I. Gupta, L. M. Steinmetz, C. Moore, T. H. Jensen, Rrp6p controls mRNA poly(A) tail length and its decoration with poly(A) binding proteins. *Mol. Cell* **47**, 267–280 (2012). doi:10.1016/j.molcel.2012.05.005 Medline

59. A. Leitner, T. Walzthoeni, R. Aebersold, Lysine-specific chemical cross-linking of protein complexes and identification of cross-linking sites using LC-MS/MS and the xQuest/xProphet software pipeline. *Nat. Protoc.* **9**, 120–137 (2014). doi:10.1038/nprot.2013.168 Medline

60. P. B. Balbo, A. Bohm, Mechanism of poly(A) polymerase: Structure of the enzyme-MgATP-RNA ternary complex and kinetic analysis. *Structure* **15**, 1117–1131 (2007). doi:10.1016/j.str.2007.07.010 Medline

61. L. S. Johnson, S. R. Eddy, E. Portugaly, Hidden Markov model speed heuristic and iterative HMM search procedure. *BMC Bioinformatics* **11**, 431 (2010). doi:10.1186/1471-2105-11-431 Medline

62. Y. Liu, B. Schmidt, D. L. Maskell, MSAProbs: Multiple sequence alignment based on pair hidden Markov models and partition function posterior probabilities. *Bioinformatics* **26**, 1958–1964 (2010). doi:10.1093/bioinformatics/btq338 Medline

63. W. Kabsch, C. Sander, Dictionary of protein secondary structure: Pattern recognition of hydrogen-bonded and geometrical features. *Biopolymers* **22**, 2577–2637 (1983). doi:10.1002/bip.360221211 Medline

64. W. G. Touw, C. Baakman, J. Black, T. A. H. te Beek, E. Krieger, R. P. Joosten, G. Vriend, A series of PDB-related databanks for everyday needs. *Nucleic Acids Res.* **43** (D1), D364–D368 (2015). doi:10.1093/nar/gku1028 Medline

65. E. Krissinel, K. Henrick, Inference of macromolecular assemblies from crystalline state. *J. Mol. Biol.* **372**, 774–797 (2007). doi:10.1016/j.jmb.2007.05.022 Medline

66. A. J. Venkatakrishnan, X. Deupi, G. Lebon, C. G. Tate, G. F. Schertler, M. M. Babu, Molecular signatures of G-protein-coupled receptors. *Nature* **494**, 185–194 (2013). doi:10.1038/nature11896 Medline

67. M. S. Cline, M. Smoot, E. Cerami, A. Kuchinsky, N. Landys, C. Workman, R. Christmas, I. Avila-Campilo, M. Creech, B. Gross, K. Hanspers, R. Isserlin, R. Kelley, S. Killcoyne, S. Lotia, S. Maere, J. Morris, K. Ono, V. Pavlovic, A. R. Pico, A. Vailaya, P.-L. Wang, A. Adler, B. R. Conklin, L. Hood, M. Kuiper, C. Sander, I. Schmulevich, B. Schwikowski, G. J. Warner, T. Ideker, G. D. Bader, Integration of biological networks and gene expression data using Cytoscape. *Nat. Protoc.* **2**, 2366–2382 (2007). doi:10.1038/nprot.2007.324 Medline

68. P. Shannon, A. Markiel, O. Ozier, N. S. Baliga, J. T. Wang, D. Ramage, N. Amin, B. Schwikowski, T. Ideker, Cytoscape: A software environment for integrated models of biomolecular interaction networks. *Genome Res.* **13**, 2498–2504 (2003). doi:10.1101/gr.1239303 Medline

69. T. Ishida, K. Kinoshita, PrDOS: Prediction of disordered protein regions from amino acid sequence. *Nucleic Acids Res.* **35** (Web Server), W460–W464 (2007). doi:10.1093/nar/gkm363 Medline

70. J. Zhao, M. Kessler, S. Helmling, J. P. O'Connor, C. Moore, Pta1, a component of yeast CF II, is required for both cleavage and poly(A) addition of mRNA precursor. *Mol. Cell. Biol.* **19**, 7733–7740 (1999). doi:10.1128/MCB.19.11.7733 Medline

71. M. A. Ghazy, X. He, B. N. Singh, M. Hampsey, C. Moore, The essential N terminus of the Pta1 scaffold protein is required for snoRNA transcription termination and Ssu72 function but is dispensable for pre-mRNA 3′-end processing. *Mol. Cell. Biol.* **29**, 2296–2307 (2009). doi:10.1128/MCB.01514-08 Medline

72. J. Chen, C. Moore, Separation of factors required for cleavage and polyadenylation of yeast pre-mRNA. *Mol. Cell. Biol.* **12**, 3470–3481 (1992). doi:10.1128/MCB.12.8.3470 Medline

73. K. Naydenova, C. J. Russo, Measuring the effects of particle orientation to improve the efficiency of electron cryomicroscopy. *Nat. Commun.* **8**, 629 (2017). doi:10.1038/s41467-017-00782-3 Medline

74. S. M. Barabino, W. Hübner, A. Jenny, L. Minvielle-Sebastia, W. Keller, The 30-kD subunit of mammalian cleavage and polyadenylation specificity factor and its yeast homolog are RNA-binding zinc finger proteins. *Genes Dev.* **11**, 1703–1716 (1997). doi:10.1101/gad.11.13.1703 Medline

75. C. Plaschka, P.-C. Lin, K. Nagai, Structure of a pre-catalytic spliceosome. *Nature* **546**, 617–621 (2017). Medline

76. M. Costanzo, B. VanderSluis, E. N. Koch, A. Baryshnikova, C. Pons, G. Tan, W. Wang, M. Usaj, J. Hanchard, S. D. Lee, V. Pelechano, E. B. Styles, M. Billmann, J. van Leeuwen, N. van Dyk, Z.-Y. Lin, E. Kuzmin, J. Nelson, J. S. Piotrowski, T. Srikumar, S. Bahr, Y. Chen, R. Deshpande, C. F. Kurat, S. C. Li, Z. Li, M. M. Usaj, H. Okada, N. Pascoe, B.-J. San Luis, S. Sharifpoor, E. Shuteriqi, S. W. Simpkins, J. Snider, H. G. Suresh, Y. Tan, H. Zhu, N. Malod-Dognin, V. Janjic, N. Przulj, O. G. Troyanskaya, I. Stagljar, T. Xia, Y. Ohya, A.-C. Gingras, B. Raught, M. Boutros, L. M. Steinmetz, C. L. Moore, A. P. Rosebrock, A. A. Caudy, C. L. Myers, B. Andrews, C. Boone, A global genetic interaction network maps a wiring diagram of cellular function. *Science* **353**, aaf1420–aaf1420 (2016). doi:10.1126/science.aaf1420 Medline
