# Supplementary material for: Architecture of eukaryotic mRNA 3′-end processing machinery
Source: Science. 2017 Oct 26;358(6366):1056–9. doi: 10.1126/science.aao6535 (PMC5788269; doi:10.1126/science.aao6535)
Supplement: Architecture of eukaryotic mRNA 3′-end processing machinery [file Science-358-1056-s3.pdf]

Additional Data S1: Sequence alignment of Pfs2 across major eukaryotic lineages with at least one representative from each lineage, constructed using MSAProbs and refined using BLAST and HMMER sequence profiles.

```
                         30        40        50        60        70        80        90
PFS2_YEAST/25-384        KYVTQRRSVDVSSPYINLYYNR....RHGLPNLVVEPETSYTIDIMPPNAYRGRD..RVINL.PSKFTHLSSNK......
PFS2_CRYNJ/63-423        RKIKPRRTVDYQGGVQKWRMLNKL.KGVHEFRPAIHPNPSDIVNFLPPVALRSN...PSTSI.CDYWVHTSINK......
A0A075ASR8_9FUNG/25-391  REAIQRRTIDYMSDLANWIENRLVC.KSAVDYPPVLNDSTYLMNMLPPSVYEDE...PINAV.CTRYIHTSVNK......
A0A1R1Y9Z7_9FUNG/1-323   ............................MKPDPSYIIDLEVPIFNKSK...GIYGV.TTRFVQQAQNK......
A0A1S8W260_9FUNG/103-460 RKAIQRRTVDYNSVLMRYWEIRNI.QRCLKDYTAMQPEPQFLVNIMPPFACSHN...LSTSV.TTKFIHLSANK......
A0A0L0T069_ALLMA/85-440  RTVDYNSGVILHNQMRTQLYPA....SRSTPWPAIQPDMEFLINLLPPYAIPDK...PVSAC.TTRFVHTSMNK......
PFS2_SCHPO/17-379        RTVDYGSGLSKYIVNRHLRSNR.......YHIHVPRPNPNQIINLYPPYEYKYNN..TSSLC..TKYIHTSANK......
A0A058Z0X0_9EUKA/80-441  SENFQRRTLDYNNSMIRYLEDRMVW.KKPGQRPAAHPDYRFEIHTMPPIAKPQH...SADLL.LSRYIYDAKNH......
WDR33_HUMAN/49-409       RKAVNRKTIDYNPSVIKYLENRIWQ.RDQRDMRAIQPDAGYYNDLVPPIGMLNN...PMNAV.TTKFVRTSTNK......
A0A1D5PT91_CHICK/49-409  RKAVNRKTIDYNPSVIKYLENRVWQ.RDQRDMRAIQPDAGYYNDLVPPIGMLNN...PMNAV.TTKFVRTSTNK......
F6RF35_XENTR/49-409      RKAVNRKTIDYNPSVIKYLENRVWQ.RDQRDMRAIQPDAGYYNDLVPPIGMINN...PLNAV.TTKFVRTSTNK......
F1QJZ5_DANRE/49-409      RKAVNRKTIDYNPSVIRHLENRLWQ.RDHRDFRAVQPDAGCYNELVPPVGMISN...PMNAV.TTKFVRTSTNK......
C3YRI5_BRAFL/1-303       ................................................MAMMEQ...PMNSV.TTKFVRTSTNK......
W4ZD79_STRPU/76-436      KNNLQRRTVDYNTSVIKYLQTRIWQ.RDYKDARMIQPDHSYIGNLSLPHQMLDN...PINAV.TTRFVRTSTNK......
Q9VNG2_DROME/76-436      RKSVMRKTVDYNASIIKALENRLYQ.RDYRDRLALQPDSIYVPHMLPPSAYLDN...PSNAV.TTRFVKTATNK......
U4PCM1_CAEEL/73-434      RKNVANVRRHVDYVSTVLNHCENRL.WQYGKQRILQQPDILYQQYAVPADSTPDV..PVDCI.LTKFIRTAMNK......
G4V905_SCHMA/33-393      RKAVFRRTIDYNASAINYLQNRIWV.SRDLDRPMLQPDYLWSPKLCPPRSYMD....SPMNCVMSKPVRTSTNK......
A7S5J4_NEMVE/9-369       RKAVIRRTIDYNSCVVKFLENRKWF.KHLDSRHGIQPDEAYAAEILPPSCMPDN...PINAV.TTKFVRTSTNK......
I1F5K4_AMPQE/421-780     SRPVIRKTVEYHATALHYLKNRLWQ.RDRRDKPLIQPDLTYYNDVSIPAGLED...CPVNVVTTKFVKACTNK......
B3RZA6_TRIAD/42-401      RKAVQRRTIDYNASVIRQLESRVWQ.SDYRDQPVIQPDPLYFNSLLPPSSMKQN...PMNAV.TTKFVRTSTNK......
A9V9K0_MONBE/87-472      TTQDHRPTIDHHAAIVADLESRLFR.KDGRDVPRLQPRDAEAYHVMPPAVYARD...CANAV.VTKFAKEGKAKSEQARA
A0A0D2WGQ4_CAPO3/114-479 RKAVVRRTVDYNASAITYLEKRIWQ.RTSRYSALLPDESFVRELELPIAYTQT...PASSV.MTKHVHSSWNK......
C7G084_DICDI/55-413      RKAVVRKTVDFNSTLIKYNLARVYQ.RDYRDFAVVQPDQLYIREILPPIALESN...PITSV.CSRFIHTSSNK......
FY_ARATH/53-411          RKHTQRRAVDYTSTVVRYIQARTWQ.RDSRDRTTLQPTPAAAVDMLPTVAYSDN...PSTSF.AAKFVHASLNK......
D5LAU9_CHLRE/30-394      QPKAIKSRVLDHVGPYLRLMQARLASPHVSHATALQPVFASSLELLPPCAYPELP...ASSV.GTKFVTTSFFR......
M2X449_GALSU/24-392      RKAVVRRTVDFNNSSMRWMLDRPGQ.ETEKDVPTLQPTAEYIPNLLPPQAPYMDNM.PISSV.CTKFVHTSINK......
A0A024V591_PLAFA/15-393  KLGIDRPAIDFTSSVCNFLKNDVYK.RQFE..RKLYVNHPIYLRRIKPLFCYSNMIDRYDGV.MSHLACSCLNK......
J9IXC5_9SPIT/4-361       QQQTQASFDFMATDTNYMHYRDFD..FDPADRPAVYPHFSYGKNIYPSSMCEDKD...WECAC..PKFLAEQENK......
A0A078APN6_STYLE/311-669 PQAASNASFDFMATDTNYQQFSEYD.FDPADRPAIYPHPSYGKYTFPAQEIYQK...DWEYA.CPKFLAEQQNR......
A0A0G4EEV8_VITBC/10-369  RRPAVREAIDFTPTVVQFFGSTIHC.RRLSDSPALLAHPSSTRDLLPPFATRES...PYDCL.CTRFLKCCVSK......
W2FMQ6_PHYPR/35-395      RKPIVRKSVDPNATFINYITQRRFVQARSVRSHALPPHSFYKKQMLPPMAYPGDDD.LASCV.CTKFCRCSTNK......
R1DYN8_EMIHU/27-391      CRKTVDYSACTVRMLKERTLCP....EREASPYTLDASPDAAVEMLPPNGTLSNA...AASA.CTRFVHSALNK......
Q4Q7R7_LEIMA/104-474     TNYNASTALCTQWVNTSYHPDSKGG.RIRTPLYALQWSPSGRRLLCSTGRGEFLL..FNGQS.FGVEVKTVAHE......
A2D7P9_TRIVA/40-403      RRFRKAISRRWCDHRQAFELYTQAR.PYNEYDVFPQNVSIGYRNLLPPFCYDT.....SGISI.CAKFIAHKNNK......
D2VL90_NAEGR/109-467     RKAIDRKFIDYSPTVIQLTRSRIYE.RSKRDLMYIPPHPAYEINLLPPSAYKQN...PTTCI.TSNFEHSSTNK......
```

```
                                    100       110       120       130       140       150
PFS2_YEAST/25-384        ...........VKHVIPAIQWTPEGRRLVVATYSGEFSLWNASSFTFETLMQAHD..SAVTTMKYSHDSDWMISGDA..
PFS2_CRYNJ/63-423        ...........ERSPTRVVRWTPDARRLLTGNDKGQFTLWNGASFNYESITQVHD..DSIRSFTYSHNGQALVSADK..
A0A075ASR8_9FUNG/25-391  ...........VRCPVNVVRWNPEGRRLITGCASGEFTLWNGFTFNFETILQAHD..SAVRCMEWSHSGNILISADH..
A0A1R1Y9Z7_9FUNG/1-323   ...........IRCPVNVVRWTPDGRRILTGSSSGEFTLWNGLTFNFETIMQAHD..TAIRGMEWSHDSQWLVSGDQ..
A0A1S8W260_9FUNG/103-460 ...........VKCPINAVRWTPEGRRLITGASSGEFTLWNGLTFNFETILQAHD..SAIRSIKWSHNDTWMISADD..
A0A0L0T069_ALLMA/85-440  ...........YRTYVNVVRWTPEGRRLITGSASGEFTLWNGLTFNFETILQAHE..TSVRSMRWSRNDTWMLSGDN..
PFS2_SCHPO/17-379        ...........ARHVINVVRWTPDGRRLLTGSSTGEFTLWNGLTFNFELINQSHD..YAVRCAEWSTDGRWLISGDG..
A0A058Z0X0_9EUKA/80-441  ...........DKYHVTSLSFLPDGRRMVTGNSHGQLTLWSSLTFNFENIVQAPAFSSPIKRLRWHPVEDTMIVGMK..
WDR33_HUMAN/49-409       ...........VKCPVFVVRWTPEGRRLVTGASSGEFTLWNGLTFNFETILQAHD..SPVRAMTWSHNDMWMLTADH..
A0A1D5PT91_CHICK/49-409  ...........VKCPVFVVRWTPEGRRLVTGASSGEFTLWNGLTFNFETILQAHD..SPVRAMTWSHNDMWMLTADH..
F6RF35_XENTR/49-409      ...........VKCPVFVVRWTPEGRRLVTGASSGEFTLWNGLTFNFETILQAHD..SPVRAMTWSHNDMWMLTADH..
F1QJZ5_DANRE/49-409      ...........VKCPVFVVRWTPEGRRLVTGASSGEFTLWNGLTFNFETILQAHD..SPVRAMTWSHNDMWMLTADH..
C3YRI5_BRAFL/1-303       ...........IRCPIFVVRWTPEGRRLVTGASSGEFTLWNGLAFNFETILQAHD..TAVRAMSWSHNDQWMVTADH..
W4ZD79_STRPU/76-436      ...........VRCPIFCVVWTPEGRRLVTGASSGEFTLWNGLTFNFETILQAHE..CAVRTMTWSRNDMWLLTGDD..
Q9VNG2_DROME/76-436      ...........MRCPIFTLAWTPEGRRLVTGASSGEFTLWNGLTFNFETILQAHD..ISVRTMVWSHNDSWMVTGDH..
U4PCM1_CAEEL/73-434      ...........VKCPVYSVCWSPEGKRLITGCQTGEFTLWNGTAFNFETILQAHD..SAIRALKWASNEQWLLSADQ..
G4V905_SCHMA/33-393      ...........NKCPIYCVCWTPEGRRLITGAFSGEFTLWNGLTFNFETILQAHE..SQIRCMKWSHNEEWLLTADH..
A7S5J4_NEMVE/9-369       ...........LRCPIFCVVWTPEGRRLVTGASSGEFTLWNGLTFNFETILTAHE..SPVRAMVWNHHDSWLLSADH..
I1F5K4_AMPQE/421-780     ...........TRSPIFSTSWVPDGRRLVTGAASGEFTLWNGINFNFESIIQAHD..CSVRTIDWSHDGKWMVTADD..
B3RZA6_TRIAD/42-401      ...........QRCPIFCVVWTPEGRRLITGASSGEFTLWNGLTFNFETILQAHD..TSVRSMIWSHNDTWLLTTDT..
A9V9K0_MONBE/87-472      QSEASAGGEALRSNTPAFCIRWDPTGRRVITGHSTGQFALWYACTFNFETILSAHF..NSIRTLNWSNDYEWLLTGDD..
A0A0D2WGQ4_CAPO3/114-479 ...........QRCPIYCAEWTPEGRRLVTGASTGEFTLWNGLTFNFETIMQAHD..TAVRAMKWSHSDSWMITADH..
C7G084_DICDI/55-413      ...........VKYPINCVSWTPEGRRLVTGSSSGEFTLWNGLTFNFEMILQAHD..SAVRSIIWSHNEDWMVSGDD..
FY_ARATH/53-411          ...........NRCSINRVLWTPSGRRLITGSQSGEFTLWNGQSFNFEMILQAHD..QPIRSMVWSHNENYMVSGDD..
D5LAU9_CHLRE/30-394      ...........HRAAINHLLYTPDGRRLLCGGNNGNVSLWNGTNFENELGPGIQA..HEASPIRCMEFSHSGLFLLS..
M2X449_GALSU/24-392      ...........VRCPINTCRWTPEGRRLITGASTGEFTLWNGFTFNFETILQAHD..SAVRTMIWSHDENWMLTGDH..
A0A024V591_PLAFA/15-393  ...........SKGMIVSLKWFNDGKRLLTGTQLSELCIWNGSYFNFEDMKRIPIGGGSVSCLEWSKNDNLF.AGNS..
J9IXC5_9SPIT/4-361       ...........QRNPLFAVQWMQGGKRLLTGSSKGDIITWFAPNYEYENATSVHR..DRVQCMCWTNNEKYLISGDK..
A0A078APN6_STYLE/311-669 ...........QGNPLFAIKWMQGGKRILTGTSKGDIITWYVPNFEYEKATSVHK..DRVQCMCWSNNERFLISGDK..
A0A0G4EEV8_VITBC/10-369  ...........SRGTVNCLQWFPDGRRLITGTSNGDFTLWDGIDFGFFDLKQLNH..SAVGALTWTHSEKTLISGNY..
W2FMQ6_PHYPR/35-395      ...........QRCPVNCLTWTPEGRRLITGNSVGEFTLWNGLAFNFETILQAHD..DAVRAMVWSHNDNWLVTADH..
R1DYN8_EMIHU/27-391      ...........DRSSVNAGCWMPDGRRLITASYNGMFTLWSGFSFNFEATQQAHD..EPLFSLCWSRGGEWLLSGDG..
Q4Q7R7_LEIMA/104-474     ...........DNRPCRAIAWGRRHDLILSGDDAGKLKMWMPNFVFMSEVDTSHR...AVREISWAPLELKFCTAGQD.
A2D7P9_TRIVA/40-403      ...........FRASTKALAWQPDSLRVVVGDQQGNITMFNGLAFFYEFHIPASN..TGIHDLIWTHSGDYLLTVDAMK
D2VL90_NAEGR/109-467     ...........ERYAVHVVSWTPDGRRLLTGISSGQFTLWNGMAFNFETIQLAHN..DPIRAMVWNHNDTFMASADN..
```

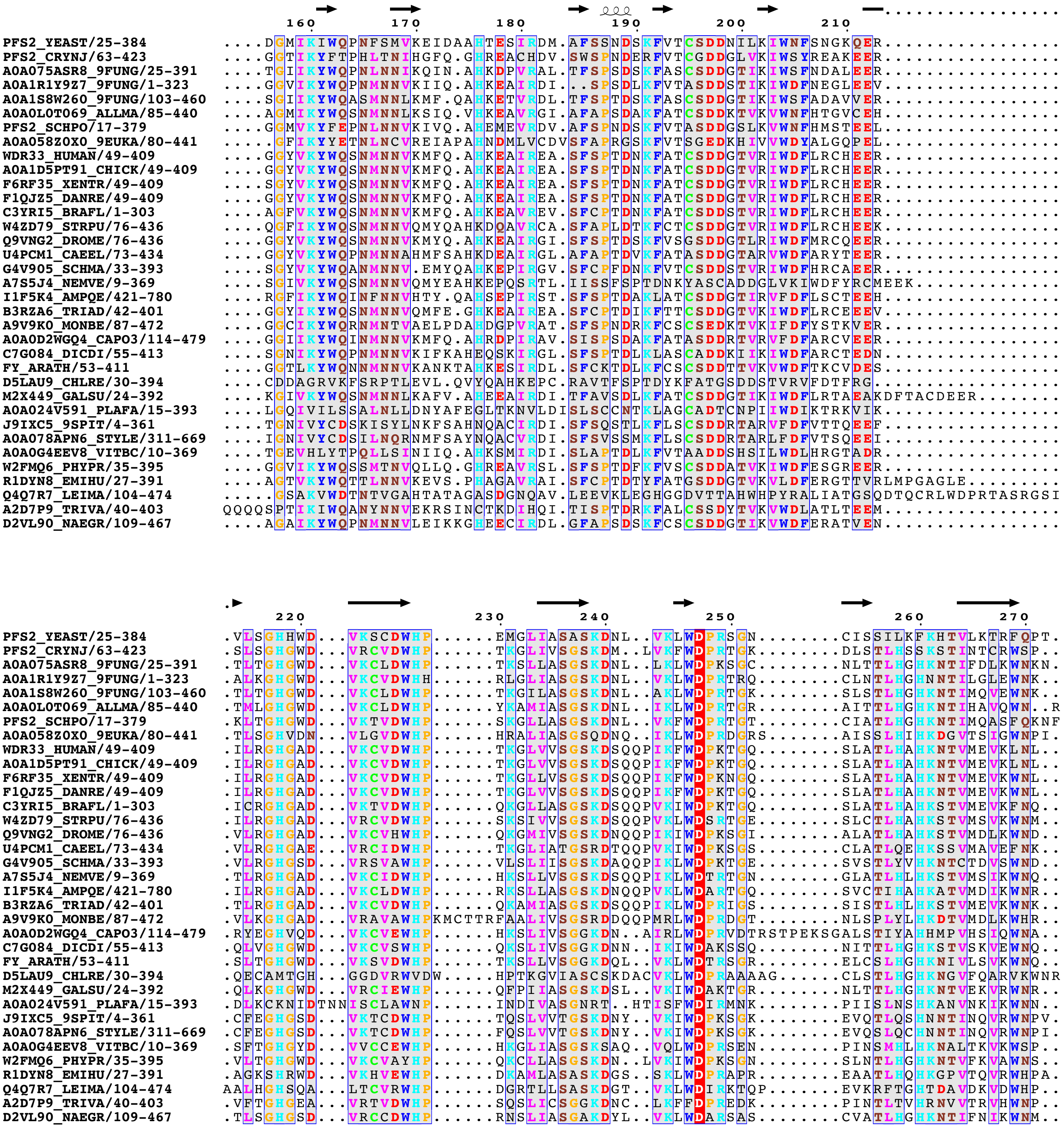
160
170
180
190
200
210
PFS2_YEAST/25-384
PFS2_CRYNJ/63-423
A0A075ASR8_9FUNG/25-391
A0A1R1Y9Z7_9FUNG/1-323
A0A1S8W260_9FUNG/103-460
A0A0L0T069_ALLMA/85-440
PFS2_SCHPO/17-379
A0A058Z0X0_9EUKA/80-441
WDR33_HUMAN/49-409
A0A1D5PT91_CHICK/49-409
F6RF35_XENTR/49-409
F1QJZ5_DANRE/49-409
C3YRI5_BRAFL/1-303
W4ZD79_STRPU/76-436
Q9VNG2_DROME/76-436
U4PCM1_CAEEL/73-434
G4V905_SCHMA/33-393
A7S5J4_NEMVE/9-369
I1F5K4_AMPQE/421-780
B3RZA6_TRIAD/42-401
A9V9K0_MONBE/87-472
A0A0D2WGQ4_CAPO3/114-479
C7G084_DICDI/55-413
FY_ARATH/53-411
D5LAU9_CHLRE/30-394
M2X449_GALSU/24-392
A0A024V591_PLAFA/15-393
J9IXC5_9SPIT/4-361
A0A078APN6_STYLE/311-669
A0A0G4EEV8_VITBC/10-369
W2FMQ6_PHYPR/35-395
R1DYN8_EMIHU/27-391
Q4Q7R7_LEIMA/104-474
A2D7P9_TRIVA/40-403
D2VL90_NAEGR/109-467
220
230
240
250
260
270
PFS2_YEAST/25-384
PFS2_CRYNJ/63-423
A0A075ASR8_9FUNG/25-391
A0A1R1Y9Z7_9FUNG/1-323
A0A1S8W260_9FUNG/103-460
A0A0L0T069_ALLMA/85-440
PFS2_SCHPO/17-379
A0A058Z0X0_9EUKA/80-441
WDR33_HUMAN/49-409
A0A1D5PT91_CHICK/49-409
F6RF35_XENTR/49-409
F1QJZ5_DANRE/49-409
C3YRI5_BRAFL/1-303
W4ZD79_STRPU/76-436
Q9VNG2_DROME/76-436
U4PCM1_CAEEL/73-434
G4V905_SCHMA/33-393
A7S5J4_NEMVE/9-369
I1F5K4_AMPQE/421-780
B3RZA6_TRIAD/42-401
A9V9K0_MONBE/87-472
A0A0D2WGQ4_CAPO3/114-479
C7G084_DICDI/55-413
FY_ARATH/53-411
D5LAU9_CHLRE/30-394
M2X449_GALSU/24-392
A0A024V591_PLAFA/15-393
J9IXC5_9SPIT/4-361
A0A078APN6_STYLE/311-669
A0A0G4EEV8_VITBC/10-369
W2FMQ6_PHYPR/35-395
R1DYN8_EMIHU/27-391
Q4Q7R7_LEIMA/104-474
A2D7P9_TRIVA/40-403
D2VL90_NAEGR/109-467

```
                                  280       290       300       310       320       330       340
PFS2_YEAST/25-384          KGNLLMAISKDKSCRVFDIRYSMKELMCVRD........ET.DYMTLEWHPINESMFTLACYDGSLKHFDLLQ.NLNEPI
PFS2_CRYNJ/63-423          DGHLVATAGQDSVIRLFDIRTFR.ELEVLKG........HEKEVNCIEWHPIHHSLLVSGDALGTINYFSLLSPTPSTPI
A0A075ASR8_9FUNG/25-391    .GNWLLTVGRDQAVKLYDIRM.MKEMQSFKG........HKKEVNALRWHPVHESLFCTSGSDGSILTWLV...GLDKPV
A0A1R1Y9Z7_9FUNG/1-323     NGWWLLSGSRDSNIQIFDIR.KMDHLRSFRG........IPKEVTSVAWHPVHENIFASGGSEGSLMMWDVS...AKDYV
A0A1S8W260_9FUNG/103-460   NGNWLLTASRDQLVRTYDIR.TMKELQVFKG........HKREVQSIRWHPVHENMFVSGGWEGSMIFWEV...GLDVPL
A0A0L0T069_ALLMA/85-440    NGNWLLSGSRDQMVKIFDIR.TMKEYANFKG........HAKEVCSVAWHPQHEDLFVSGGSEGSLIWWLAS..QPSIPL
PFS2_SCHPO/17-379          GSNYLATVSRDSTCRVFDLR.AMKDVRVLRG........HEKDVNCVTWHPLYPNLLTTGGSDGSVNHYSLDE.PPLLSQ
A0A058Z0X0_9EUKA/80-441    NGNWLASTSRDSTAIIYDIRA.MDVLQTFSL........AD.RVSSLAWHPLREDILATGCMNGTLA.FSLI..GLEEPH
WDR33_HUMAN/49-409         NGNWLLTASRDHLCKLFDIRNLKEELQVFRG........HKKEATAVAWHPVHEGLFASGGSDGSLLFWHV...GVEKEV
A0A1D5PT91_CHICK/49-409    NGNWLLTASRDHLCKLFDIRNLKEELQVFRG........HKKEATAVAWHPVHEGLFASGGSDGSLLFWHV...GVEKEV
F6RF35_XENTR/49-409        NGNWLLTASRDHLCKLFDIRNLREELQVFRG........HKKEATAVAWHPVHEGLFASGGSDGSLLFWHV...GVEKEV
F1QJZ5_DANRE/49-409        NGNWLLTASRDHLCKLFDIRNLKEELQVFRG........HKKEATAVAWHPVHEGMFASGGSDGSLLFWHV...GVEKEV
C3YRI5_BRAFL/1-303         NGNWLLTASRDHLVKVFDIR.TMREMQSFRG........HKKEATAVAWHPIHESLFASGGSEGSILFWQV...GNDKEV
W4ZD79_STRPU/76-436        NGNWLLTASRDHLVKLFDIRMMK.ELFTFRG........HKKEATSIAWHPIHEDMFVSGGSDGGILFWQV...GHDKEI
Q9VNG2_DROME/76-436        NGNWLVTASRDHLLKLFDIRNLREEVQVFRG........HKKEASSVSWHPIHEGLFCSGGSDGSILFWNV...GTDKEI
U4PCM1_CAEEL/73-434        NGNWLLTGGRDHLVKMYDIRMMK.EMRTYRA........HKKEVISLAWHPIHEGLFVSGGGDGSIVYWMV...DGEKEI
G4V905_SCHMA/33-393        NGNWFLTASRDHLIKLFDLRNLKSELQTFRGH.......KR.DVMRVAWHPFHESLFVSGSADGAIFYWLA...GTDTEL
A7S5J4_NEMVE/9-369         NGNWLLTASRDHLLKLFDIR.AMKELQAFRG........HKREATAIAWHPIHENLFASGGSDGAIMFWMS...GSDKDV
I1F5K4_AMPQE/421-780       NGNWLATASRDYLIKLYDVRM.MKELYVLKGH.......KK.DVNTISWHPVHEKVFASGGSDGAILFWLV...GNEREI
B3RZA6_TRIAD/42-401        NGNWLLTASRDHLLKIFDIR.AMKELQTFRG........HKKEATAVAWHPVHEGLFASGGSDGAMYFWIA...GCDKEV
A9V9K0_MONBE/87-472        DGNMLLSASRDGLVKLFDLRTMK.EMQTFRA........HEGQVDVVAWHPIHNDLFVSSSGKGGLHFWLAG..TSSALG
A0A0D2WGQ4_CAPO3/114-479   NGNWFISGSRDHQIKVYDIRVMK.EMQTFRG........HKMDIDSLSWHPVHEELFASGGSDGSLMYWMV...GVDECL
C7G084_DICDI/55-413        NGNWIVSASSDQLLKVFDIR.TMKEMQTFKG........HGKEVTALALHPYHEDLFVSGDKDGKILYWIV...GNPEPQ
FY_ARATH/53-411            NGNWLLTASKDQIIKLYDIR.TMKELQSFRG........HTKDVTSLAWHPCHEEYFVSGSSDGSICHWIV...GHENPQ
D5LAU9_CHLRE/30-394        NGHWLLSCSRDQLVKLYDVRM.LKEVASFAG........HGRDVACVAWHPQHEELFVSGAGDGSLMMWLA...SRPDAQ
M2X449_GALSU/24-392        NGYWLATCGRDQLIKLYDIRMM.QELQTFRG........HKREVTSCQWHPYHETLFVSGGWDCCIFFWLV...GANEPA
A0A024V591_PLAFA/15-393    NGIYLLSCSKDSLIKLWDIRNFKLLYSYKNDQIQNNKFTSNYEPTYIAWNPIQNHIFSSADNKGNIKFYST...NDNKCI
J9IXC5_9SPIT/4-361         NGNWLLTGSRDSTLKVHDIR.TMREFNVFQK........HDNQVTVVAWHPFKEELFVSASYSGQIIYWQA....NSGHL
A0A078APN6_STYLE/311-669   NGNWLLTGSRDLSLKVYDIRTMKE.FNVFQR........HSDQVTVIAWHPFKEELFASASHNGEIIYWQA....NSGHL
A0A0G4EEV8_VITBC/10-369    SGDYLLTCGKDQSIKVVDLRTLSV.AHTLRG........HQADVTAAAWHPYHQSIVATGDYGGHLAYWDVSD.PSGSPM
W2FMQ6_PHYPR/35-395        NGNWLLTASRDQLIKIYDIRMLKE.FATLKG........HAREVTSIAWHPWYERLFVSGSYDGSLMYWEV...GKENAL
R1DYN8_EMIHU/27-391        GRYLLSGSKDQLTRLSDEAAAFKG....HKR...........EVSALSWHPVHAELFASGCHGGAV.HFWSTT.RPEEPL
Q4Q7R7_LEIMA/104-474       TVSDLFASTGADGSVMYWMVAEGDGTMTHGV........EEVVHDAAMIEAAHEKFRDKPNPVHCVAWSPL...GNILAS
A2D7P9_TRIVA/40-403        NGNLLASCGRDSMVYITDVRTMSPIAECKGH........ENDVFAVRWHPIREDLLVSAGYKGDIL.WWTT...GSNEPI
D2VL90_NAEGR/109-467       NGNWVMSCSKDQLIKLYDIRY.LKEFQTFKG........HNREVTCLAWHPFHENLFASGSYDGNIMFWMV...GNESPQ
```

```
                                        350                 360       370       380
PFS2_YEAST/25-384          LTI......PYAHDKCITSLSY.........NPVGHIFATAAKDRTIRFWTRARPID
PFS2_CRYNJ/63-423          TTL......SAAHEDAVFSLSF.........HPLGHILCSGSKDFTARFWCRARPPG
A0A075ASR8_9FUNG/25-391    GVL......EEAHDQAIWTLDWYIFSCLNNRHPVGHLLVSGSNDHCARFWARHRPQD
A0A1R1Y9Z7_9FUNG/1-323     HLI......PTAHESYVWSIDW.........HPIGHLLASGSNDHTTRFWARSRPGD
A0A1S8W260_9FUNG/103-460   AVM......ESAHDNSVYSLDW.........HPLGHMLVSGSNDHTTRWWTRNRPGD
A0A0L0T069_ALLMA/85-440    AQV......PTAHDANIWALDW.........HPLGHILCTGSNDYATRFWTRPRPGE
PFS2_SCHPO/17-379          QKY.....HEKHPNVTLSASSY..LLYPTAEIPFAHDLGIWSMQYHPLGHLLCTGS.
A0A058Z0X0_9EUKA/80-441    VQI......VRAHDRDISTLSW.........HPLGHMLASGSNDFLCKFWSLSIPGD
WDR33_HUMAN/49-409         GGM......EMAHEGMIWSLAW.........HPLGHILCSGSNDHTSKFWTRNRPGD
A0A1D5PT91_CHICK/49-409    GGM......EMAHEGMIWSLAW.........HPLGHILCSGSNDHTSKFWTRNRPGD
F6RF35_XENTR/49-409        GGM......EIAHEGMIWSLAW.........HPLGHILCSGSNDHTSKFWTRNRPGD
F1QJZ5_DANRE/49-409        GGM......EMAHEGMIWSLAW.........HPLGHILCSGSNDHTSKFWTRNRPGD
C3YRI5_BRAFL/1-303         GGM......ENAHEGMIWSLAW.........HPLGHILCSGSNDHST...........
W4ZD79_STRPU/76-436        GGI......EEAHESIIWSLAW.........HPLGHMLCSGSNDHSTKFWTRNRPGD
Q9VNG2_DROME/76-436        GCV......ETAHDSIVWTLAW.........HPLGHILCSGSNDHTIKFWTRNRPGD
U4PCM1_CAEEL/73-434        GLL......EHAHDQAIWSMKW.........HPLGHILATGSNDNNTKFWARNRPGD
G4V905_SCHMA/33-393        GAV......DNAHESMIWSLAW.........HPFGHILVSGANDFATKFWTRNRPGD
A7S5J4_NEMVE/9-369         GNM......ESAHEGMVWSLAW.........HPLGHILCSGSNDHTSKFWTRNR...
I1F5K4_AMPQE/421-780       GGM......ESAHEGFVWSLAW.........HPLGHLLCSGANDHTCKFWCRNRPGE
B3RZA6_TRIAD/42-401        GGI......EQAHEGMIWSLAW.........HPLGHILCSGSNDHSSKFWTRNRPGD
A9V9K0_MONBE/87-472        SIP......NAHNDAFIWDLAW.........HPLGHVLATAGNDHRVAFWTRHMPGD
A0A0D2WGQ4_CAPO3/114-479   GAV......ENAHDGNIWTLTW.........HPLGHVICSASNDHSTKFWTRPRPGE
C7G084_DICDI/55-413        AEI......HSAHDADVWSLSW.........HPIGHILASGSNDHTTKFWSRNRPAD
FY_ARATH/53-411            IEI......PNAHDNSVWDLAW.........HPIGYLLCSGSNDHTTKFWCRNRPAD
D5LAU9_CHLRE/30-394        GCL......PAAHEAAVWSAAW.........HPLGHVLASAGGDNKCQFWCRKRPGD
M2X449_GALSU/24-392        AVI......PDAHESAVWDLNW.........HPIGHILVSSSNDHATKFWTRNRPGD
A0A024V591_PLAFA/15-393    QTI......VSAHGIDNKISSISLLDW....NPLGHILTSFGDDKLLKFWSTSSSGS
J9IXC5_9SPIT/4-361         HTI......NNAHNNMIWGMAW.........HPQGQMLASTGNDHKIRFWGRNKPNE
A0A078APN6_STYLE/311-669   HTI......SKAHTAMVWGMAW.........HPQGHMLASTSNDHKIRLWGRNKPNE
A0A0G4EEV8_VITBC/10-369    ATV......PNAHDHCVLSLAW.........HPLGPIVASGGQDCTTRFWGRSLPGD
W2FMQ6_PHYPR/35-395        ASI......PQAHDTAVWDIQW.........HPVGHVVATGSNDHSTKFWCRNRPGD
R1DYN8_EMIHU/27-391        SSCHGHGHASNAHDSAVWGVEW.........HPLGHLLASFSQDMHTKFWSRARPGE
Q4Q7R7_LEIMA/104-474       SGH......EVKYWTRN...............KPGALE..EKERGVESDILDEQGHV
A2D7P9_TRIVA/40-403        YSR......MKGHNKAVFQLKF.........NPMGTILASTGTEGVVKFWVRNMCGT
D2VL90_NAEGR/109-467       AKI......KEAHSSAVWDIAW.........HPFGHILCSGSNDHSTKFWVRNRPGD
```

UniprotID	Organism
PFS2_YEAST	*Saccharomyces cerevisiae (strain ATCC 204508 / S288c) (Baker's yeast)*
PFS2_CRYNJ	*Cryptococcus neoformans var. neoformans serotype D*
A0A075ASR8_9FUNG	*Rozella allomycis CSF55*
A0A1R1Y9Z7_9FUNG	*Smittium culicis*
A0A1S8W260_9FUNG	*Batrachochytrium salamandrivorans*
A0A0L0T069_ALLMA	*Allomyces macrogynus ATCC 38327*
PFS2_SCHPO	*Schizosaccharomyces pombe (strain 972 / ATCC 24843) (Fission yeast)*
A0A058Z0X0_9EUKA	*Fonticula alba*
WDR33_HUMAN	*Homo sapiens (Human)*
A0A1D5PT91_CHICK	*Gallus gallus (Chicken)*
F6RF35_XENTR	*Xenopus tropicalis (Western clawed frog) (Silurana tropicalis)*
F1QJZ5_DANRE	*Danio rerio (Zebrafish) (Brachydanio rerio)*
C3YRI5_BRAFL	*Branchiostoma floridae (Florida lancelet) (Amphioxus)*
W4ZD79_STRPU	*Strongylocentrotus purpuratus (Purple sea urchin)*
Q9VNG2_DROME	*Drosophila melanogaster (Fruit fly)*
U4PCM1_CAEEL	*Caenorhabditis elegans*
G4V905_SCHMA	*Schistosoma mansoni (Blood fluke)*
A7S5J4_NEMVE	*Nematostella vectensis (Starlet sea anemone)*
I1F5K4_AMPQE	*Amphimedon queenslandica (Sponge)*
B3RZA6_TRIAD	*Trichoplax adhaerens (Trichoplax reptans)*
A9V9K0_MONBE	*Monosiga brevicollis (Choanoflagellate)*
A0A0D2WGQ4_CAPO3	*Capsaspora owczarzaki (strain ATCC 30864)*
C7G084_DICDI	*Dictyostelium discoideum (Slime mold)*
FY_ARATH	*Arabidopsis thaliana (Mouse-ear cress)*
D5LAU9_CHLRE	*Chlamydomonas reinhardtii (Chlamydomonas smithii)*
M2X449_GALSU	*Galdieria sulphuraria (Red alga)*
A0A024V591_PLAFA	*Plasmodium falciparum Vietnam Oak-Knoll (FVO)*
J9IXC5_9SPIT	*Oxytricha trifallax*
A0A078APN6_STYLE	*Stylonychia lemnae (Ciliate)*
A0A0G4EEV8_VITBC	*Vitrella brassicaformis (strain CCMP3155)*
W2FMQ6_PHYPR	*Phytophthora parasitica (Potato buckeye rot agent)*
R1DYN8_EMIHU	*Emiliania huxleyi (Pontosphaera huxleyi)*
Q4Q7R7_LEIMA	*Leishmania major*
A2D7P9_TRIVA	*Trichomonas vaginalis*
D2VL90_NAEGR	*Naegleria gruberi (Amoeba)*
